# Different germline variants in the *XPA* gene are associated with severe, intermediate, or mild neurodegeneration in xeroderma pigmentosum patients

**DOI:** 10.1371/journal.pgen.1011265

**Published:** 2024-12-02

**Authors:** Jeffrey P. Sagun, Sikandar G. Khan, Kyoko Imoto, Deborah Tamura, Kyu-Seon Oh, John J. DiGiovanna, Kenneth H. Kraemer

**Affiliations:** 1 Laboratory of Cancer Biology and Genetics, DNA Repair Section, Center for Cancer Research, National Cancer Institute, National Institutes of Health, Bethesda, Maryland, United States of America; 2 Nara Medical University, Kashihara, Japan; 3 Laboratory of Molecular Biology and Immunology, National Institute on Aging, National Institutes of Health, Baltimore, Maryland, United States of America; Hasselt University: Universiteit Hasselt, BELGIUM

## Abstract

Xeroderma pigmentosum (XP) is a rare autosomal recessive disease caused by pathogenic variants in seven nucleotide excision repair genes (XPA to XPG) and POLH involved in translesion synthesis. XP patients have a >1000-fold increased risk for sunlight-induced skin cancers. Many Japanese XP-A patients have severe neurological symptoms due to a founder variant in intron 3 of the *XPA* gene. However, in the United States we found XP-A patients with milder clinical features. We developed a simple scoring scale to assess XP-A patients of varying neurological disease severity. We report 18 XP-A patients examined between 1973 and 2023 under an IRB approved natural history study. Using our scale, we classified our XP-A cohort into severe (n = 8), intermediate (n = 5), and mild (n = 5) disease groups at age 10 years. DNA repair tests demonstrated greatest reduction of DNA repair in cells from severe patients as compared to cells from mild patients. Nucleotide sequencing identified 18 germline pathogenic variants in the 273 amino acid, 6 exon-containing *XPA* gene. Based on patient clinical features, we associated these *XPA* variants to severe (n = 8), intermediate (n = 6), and mild (n = 4) clinical phenotypes in the patients. Protein structural analysis showed that nonsense and frameshift premature stop codon pathogenic variants located in exons 3 and 5 correlated with severe disease. Intermediate disease correlated with a splice variant at the last base in exon 4. Mild disease correlated with a frameshift variant in exon 1 with a predicted re-initiation in exon 2; a splice variant that created a new strong donor site in intron 4; and a large genomic deletion spanning exon 6. Our findings revealed correlations between disease severity, DNA repair capacity, and *XPA* variant type and location. In addition, both XPA alleles contributed to the phenotypic differences in XP-A patients.

## Author summary

Xeroderma pigmentosum, (XP), is a rare autosomal recessive inherited disease. Exposure to ultraviolet radiation greatly increases the risk of developing skin cancers and premature aging features in XP patients. In Japan, most XP-A patients have a founder variant in intron 3 of the *XPA* gene and experience severe neurological symptoms and death at an early age. However, in our cohort we identified different germline variants in the *XPA* gene associated with severe and mild clinical features in the patients. To assess XP-A patients with a wider range of disease severity, we developed a simple neurological scoring scale. Using an age of evaluation of 10 years, we categorized phenotypic differences among our 18 XP-A patients and classified them into severe, intermediate, and mild disease groups. Using this scale, we associated these 18 pathogenic variants with severe (n = 8), intermediate (n = 6), and mild (n = 4) clinical phenotypes in the XP-A patients. As a result, our scale helps predict neurological severity and mortality, making it a useful tool for general clinical practice and genotype-phenotype studies. This allowed us to correlate neurological severity with the type, location, and cellular function of *XPA* germline pathogenic variants.

## Introduction

Xeroderma pigmentosum (XP) is a rare autosomal recessive disorder caused by pathogenic variants in seven DNA nucleotide excision repair (NER) genes, *XPA* to *XPG*, and the *POLH* gene (XP variant), which is involved in translesion DNA synthesis [1–3]. These germline variants result in impaired ability of cells to repair ultraviolet radiation (UV)-induced DNA damage. This leads to the accumulation of DNA lesions, which results in a high frequency of somatic variants or cell death, causing a range of clinical features such as hypersensitivity to UV, premature aging of the skin, and a greatly increased risk (>1,000-fold) of developing skin cancers, including basal cell carcinoma, squamous cell carcinoma, and melanoma [4–6].

XP complementation group A (XP-A) is caused by germline variants in the *XPA* gene, which encodes for the XPA scaffolding protein, a key component of the NER pathway [7,8]. Neurological abnormalities, such as progressive neurodegeneration, developmental delay, walking impairment, sensorineural hearing loss, and brain atrophy, are commonly observed in XP-A patients. The prevalence of XP varies among different populations, with estimated frequencies of 1 in 1,000,000 in the United States and Europe compared to 1 in 22,000 in Japan [9]. Many Japanese XP-A patients carry the homozygous IVS3-1G>C splice acceptor founder variant in intron 3 of the *XPA* gene. These patients exhibit a severe clinical phenotype with an early onset of pronounced neurological manifestations and death by age 20 years [10–12].

We extensively studied all XP-A patients admitted to the NIH between 1971–2023 and evaluated their medical records from NIH and external medical facilities. Surprisingly, unlike the Japanese experience, we identified XP-A patients with severe, intermediate, and mild disease phenotypes. We developed a simple, reproducible scale that could be applied to XP patients with a broad spectrum of neurological disease to assist in prediction of disease severity and mortality. We performed DNA nucleotide sequencing to identify *XPA* germline variants, performed DNA repair related functional tests and Western blotting to demonstrate whether DNA repair capacity and the message level correlates with disease severity, and compared our results to those previously reported in the literature [13–17].

## Results and discussion

### Patients and clinical assessment methods

**Fig 1:**
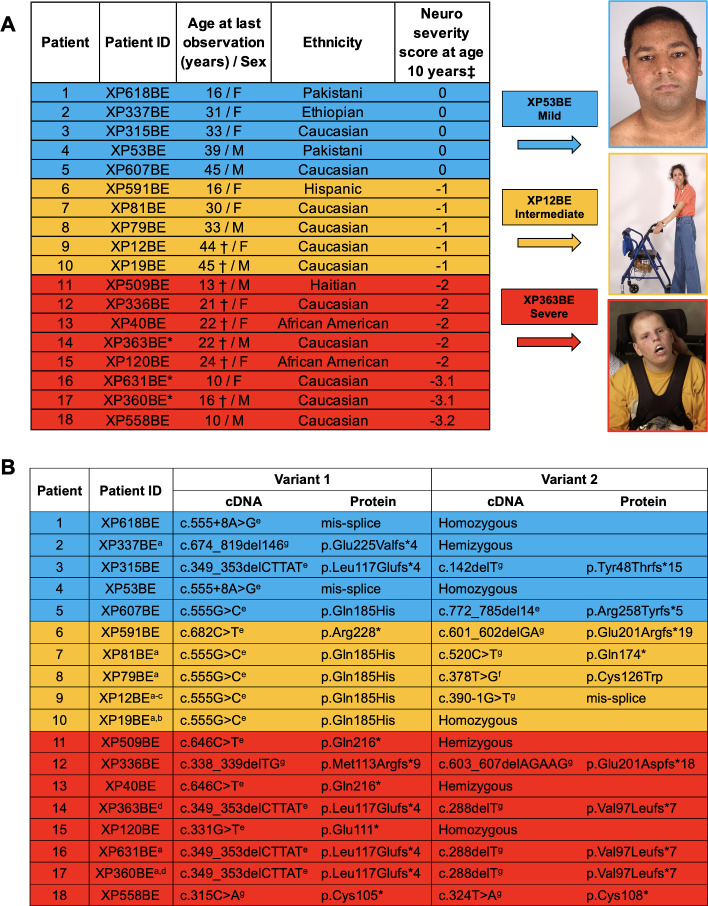
Clinical features and germline variants in 18 NIH XP-A patients from 16 families. (A) Demographic, ethnic, and clinical neurological features of 18 NIH XP-A patients. Patients are classified into three severity groups (mild (blue), intermediate (gold), and severe (red)) based on the extent of their neurological abnormalities at age 10 years. Images of patients XP53BE (age 21, mild), XP12BE (age 37, intermediate) [41], and XP363BE (age 17, severe) [32] are presented with permission from their families. *Members of the same family †Death; M: Male; F: Female; ‡Neurological severity score is based on Table 1. (B) *XPA* germline variants identified in 18 NIH XP-A patients. The references associated with patient IDs indicates the clinical descriptions of the patients. Patients were reported in references ^a^Ref [12], ^b^Ref [41], ^c^Ref [42], and ^d^Ref [32]. All other patients presented in this study are newly reported. Variants were previously reported in XP12BE, XP363BE, and XP360BE. Variant severity was categorized as mild (blue), intermediate (gold), and severe (red) based on the patients’ clinical neurologic features in Fig 1A. Variants were classified by the Human Gene Mutation Database as ^e^pathogenic, ^f^conflicting interpretations of pathogenicity, or ^g^not listed on database.

XP-A patients and their families in our natural history study were referred to the National Institutes of Health (USA) for evaluation under protocol 99C0099, approved by the National Cancer Institute Institutional Review Board. Written informed consent was obtained from all families. All enrolled patients underwent evaluation at NIH and remotely between 1971 and 2023. NIH examinations included a complete medical history, physical exam, laboratory testing, and the following examinations as appropriate: magnetic resonance imaging (MRI), computed tomography (CT) scan, dual-energy x-ray absorptiometry (DEXA) scan, thyroid ultrasound, nerve conduction study, DNA repair testing, clinical photography, speech therapy, swallowing studies, and consultations from ophthalmology, audiometry, gynecology, rehabilitation medicine, occupational therapy, and neurology services [5,18–22]. Patients were remotely followed up through telehealth visits, emails, and phone calls. Medical records from outside institutions were obtained. Patients received treatment from their local healthcare providers. In this study, the patients’ age was determined based on the most recent clinical data available. We identified 18 XP-A patients in our cohort. We described the demographic, ethnic, and clinical neurological features of our patient cohort in Fig 1.

### Neurological assessment scoring scale and classification

We initially utilized a scoring scale [12], which distinguished Japanese XP-A patients based on walking impairment and developmental delay, as assessed by intelligence quotient (IQ) scores. However, almost all the Japanese XP-A patients had severe neurological involvement. Since we had XP-A patients with varying degrees of disease severity in our cohort, we modified this scale to develop a simple, reproducible neurological assessment scoring scale (Table 1) based on the clinical symptoms observed in our patient cohort. We studied 18 XP-A patients (10 females and 8 males) from 16 different unrelated families.

**Table 1:** Neurological abnormality severity scoring scale of XP-A patients^1^.

Major Features*	
Score	Neurological Abnormality Symptoms
0	No symptoms
-0.5	Delayed developmental milestones or neurocognitive delays in early childhood Hearing decline (no hearing aid required)
-1	Mild developmental delay (IQ of 50–85), only hyporeflexia Mild hearing loss (4F-PTA or SRT of 20–40 dBHL; wears hearing aids)
-1.5	Moderate hearing loss (4F-PTA or SRT of 40–70 dBHL; wears hearing aids)
-2	Gait disturbance due to spasticity or ataxia Moderate developmental delay (IQ of 35–50)
-2.5	Severe hearing loss (4F-PTA or SRT of 70–95 dBHL; wears hearing aids)
-3	Cannot walk Severe developmental delay (IQ<35) G-tube Profound hearing loss (4F-PTA or SRT >95 dBHL; wears hearing aids)
-4	Deceased from neurological degeneration
**Minor Features‡**	
-0.1	Peripheral neuropathy (motor or sensory)
-0.1	Progressive dysphagia
-0.1	History of seizures

^1^Modified from Nishigori et al., Gene Alterations and Clinical Characteristics of Xeroderma Pigmentosum Group A Patients in Japan, Archives of Dermatology, 1994 [12]

*Major scoring criteria assigns scores based on the presence of specific major neurological symptoms present. **‡**Minor scoring criteria are additive and contribute a score of “-0.1” to the major score for each minor neurological symptom present. **IQ:** Intelligence Quotient **4F-PTA:** four-frequency pure-tone average (0.5/1/2/4 kHz) **SRT:** speech reception threshold **dBHL:** decibels hearing loss.

Neurological features were evaluated in our patients and incorporated into the scale. We divided our scale into major and minor neurological features. Major features included developmental delay, gait disturbance, mild to profound hearing loss with or without the requirement of a hearing aid [18], and gastrostomy tube (G-tube) dependency. Minor features included peripheral neuropathy (motor or sensory), progressive dysphagia, and a history of seizures. Neurological abnormality scores ranged from no symptoms (score of 0) to death due to neurological degeneration (score of -4). These additions, which incorporated more objective data, provided a simple, yet more comprehensive neurological assessment for our patient cohort.

**Fig 2:**
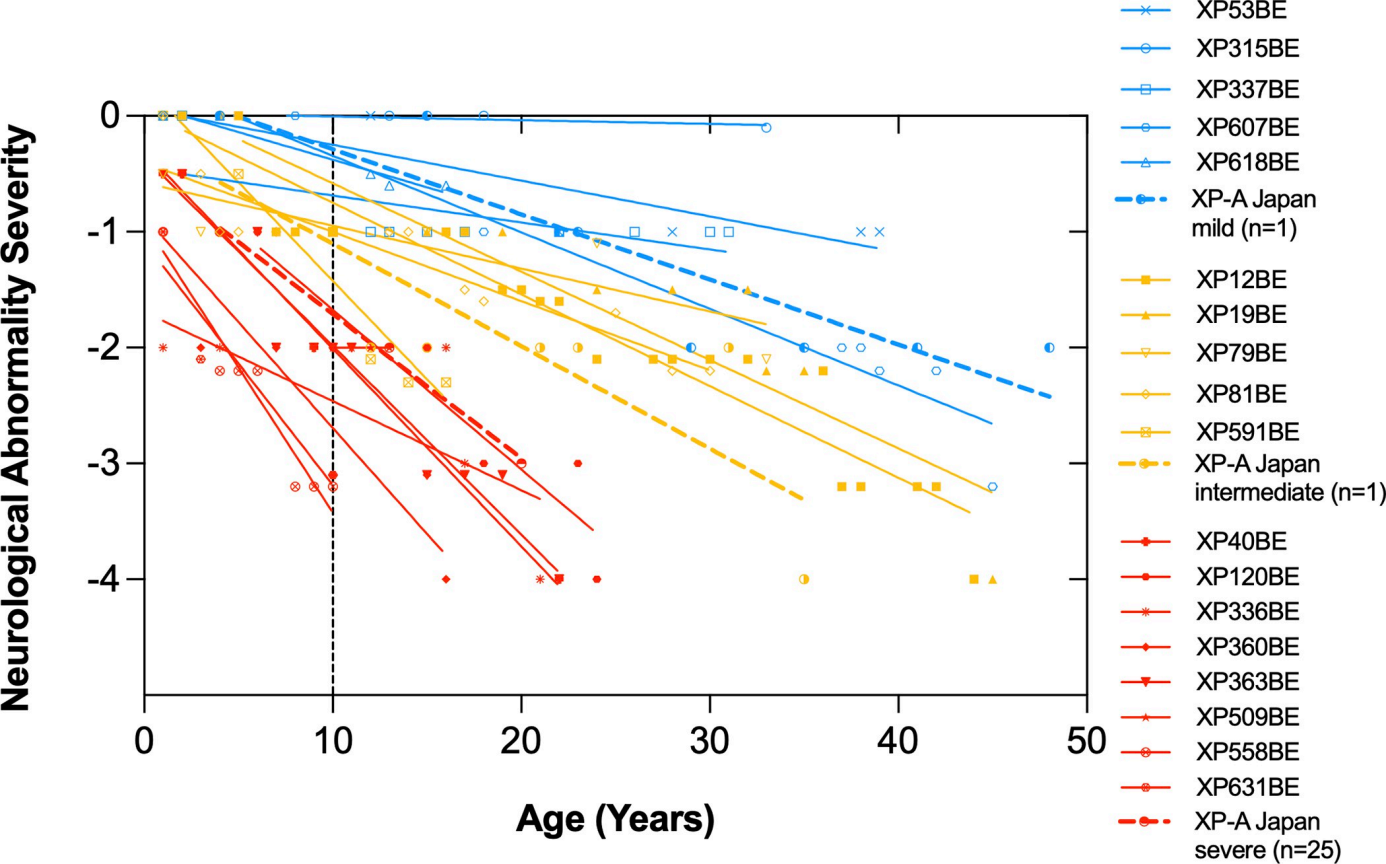
Neurological degeneration and survival of 18 NIH XP-A patients. Neurological abnormality severity scores (Table 1) are plotted against patients’ age. Solid colored lines represent severe (red) (n = 8), intermediate (gold) (n = 5), and mild (blue) (n = 5) NIH XP-A patients. Black dotted line represents age 10 years. Open symbols indicate living patients. Closed symbols indicate deceased patients. Dotted colored lines represent, severe [12], intermediate [93,94], and mild [93,94] XP-A Japanese patients reported previously. Evaluation at age 10 years was used to classify the three groups. Y-axis was displaced for clarity in XP618BE patient data set.

When using this scale, our patients showed progressive neurological decline and differences in the severity of neurological abnormalities at different ages. This prompted us to investigate the earliest age for stratifying our patient cohort. We found that using age of evaluation of 10 years would permit us to separate patients into mild (score of 0), intermediate (score of -1), or severe disease (score of -2 or lower). Using this scale, we classified 5 patients as mild, 5 patients as intermediate, and 8 patients as severe (Figs 1 and 2).

**Fig 3:**
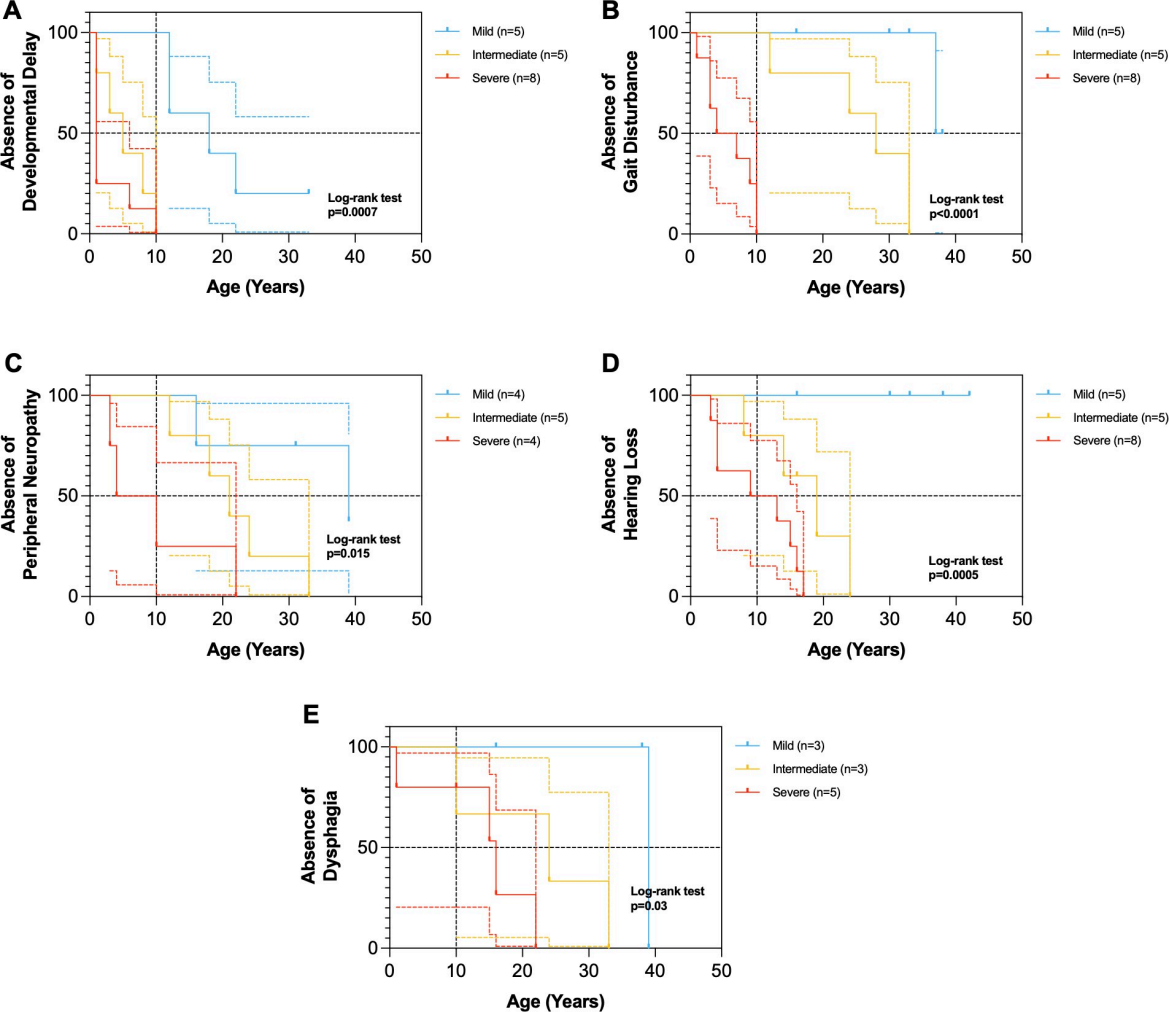
Age of onset of neurological abnormality symptoms in NIH XP-A patients. Colored dotted lines represent 95% CI. Horizontal black dotted line represents the median age of neurological abnormality symptom (see Table 1). Vertical black dotted line represents at age 10 years. (A) Kaplan-Meier plot for developmental delay in 18 patients (χ2 = 14.54, df = 2, p = 0.0007). Median age of severe group (n = 8) was 1 year, intermediate (n = 5) was 5 years, and mild (n = 5) was 18 years. (B) Kaplan-Meier plot for gait disturbance in 18 patients (χ2 = 22.92, df = 2, p<0.0001). Median age of severe group (n = 8) was 5.5 years, intermediate (n = 5) was 28 years, and mild (n = 5) was 37.5 years. (C) Kaplan-Meier plot for peripheral neuropathy in 13 patients (χ2 = 8.44, df = 2, p = 0.015). Median age of severe group (n = 4) was 7 years, intermediate (n = 5) was 21 years, and mild (n = 4) was 39 years. (D) Kaplan-Meier plot for hearing loss in 18 patients (χ2 = 15.17, df = 2, p = 0.0005). Median age of severe group (n = 8) was 11 years, intermediate (n = 5) was 19 years, and mild (n = 5) had no hearing loss. (E) Kaplan-Meier plot for dysphagia in 11 patients (χ2 = 7.1, df = 2, p = 0.03). Median age of severe group (n = 5) was 16 years, intermediate (n = 3) was 24 years, and mild (n = 3) was 39 years.

At age 10, Kaplan-Meier analyses revealed significant differences in the age of onset of developmental delay (p = 0.0007), gait disturbance (p<0.0001), peripheral neuropathy (p = 0.015), hearing loss (p = 0.0005), and dysphagia (p = 0.03) among mild patients, intermediate patients, and severe patients (Figs 3 and S1).

**Fig 4:**
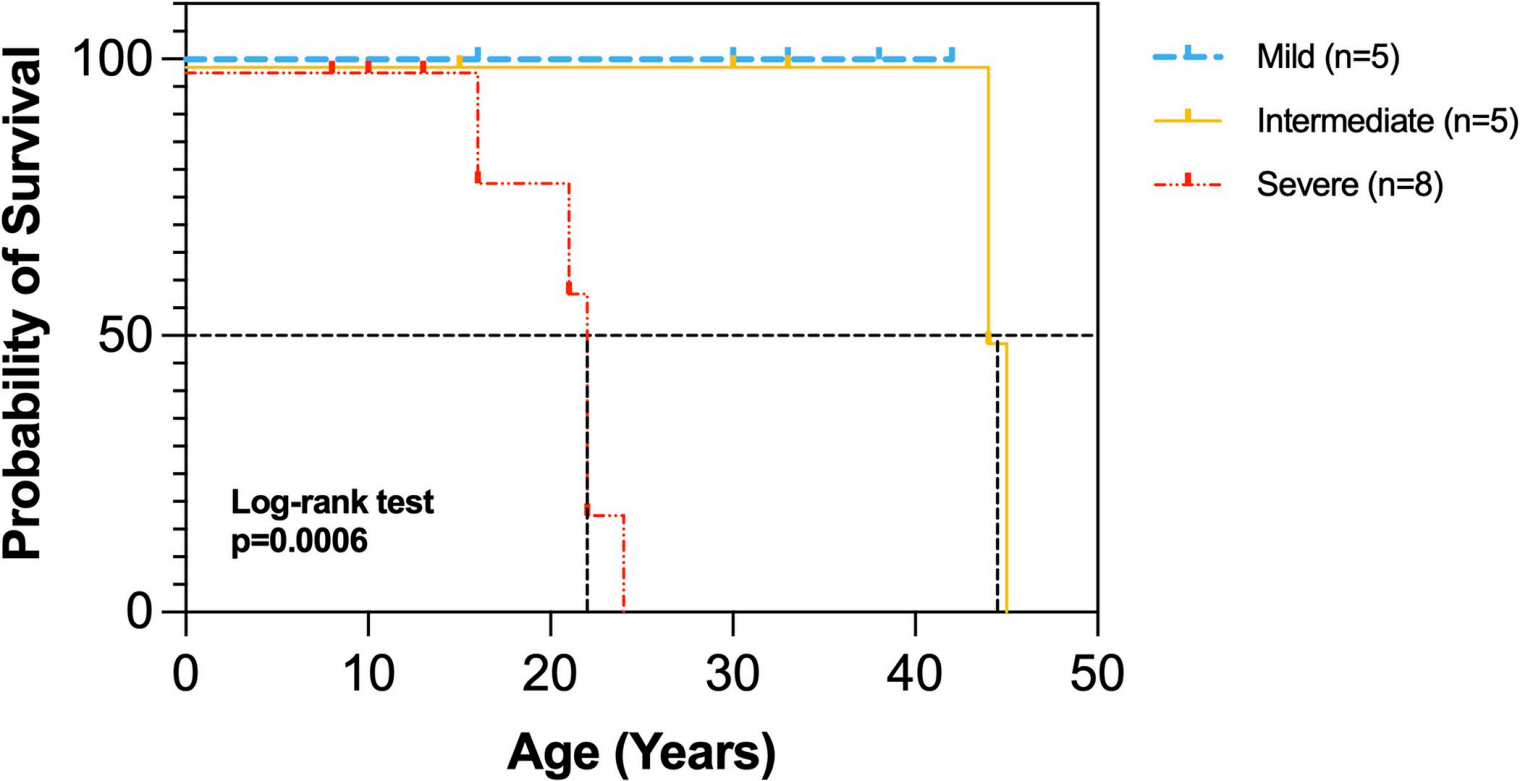
Kaplan-Meier survival curves of 18 NIH XP-A patients. Black dotted lines represent the median age of survival. Median survival of severe patients (n = 8) was 22 years, intermediate patients (n = 5) was 44.5 years, and all mild patients (n = 5) were living (χ2 = 14.7, df = 2, p = 0.0006). Y-axis was displaced for clarity in severe and intermediate patient data sets.

Survival analysis revealed a significant difference in the median age of survival among the three severity groups (p = 0.0006) (Fig 4). All mild patients were alive at the time of analysis. The median age of death for intermediate patients was 44.5 years and for severe patients was 22 years. These data suggest that early assessment of neurological abnormalities in XP-A patients at age 10 years may provide insights into their future risk for progressive neurodegeneration and death.

### Characterization of 18 *XPA* germline variants

**Fig 5:**
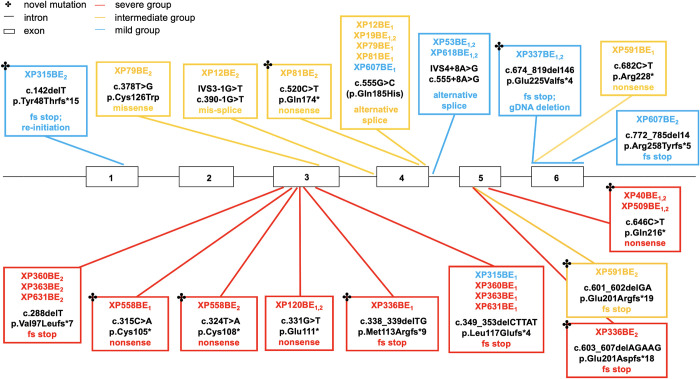
Distribution of 18 variants in the *XPA* gene in 18 NIH XP-A patients. Linear map of *XPA* germline variants associated with clinical neurological severity groups: severe (n = 8), intermediate (n = 7), and mild (n = 4). Asterisk (✤) indicates novel variants. Horizontal black lines represent introns. Black boxes represent exons. Subscripts indicates alleles. Colored patient ID indicate the clinical severity of the patient (severe (red), intermediate (gold), and mild (blue)) (see Fig 1). The colored outline of the box represents the clinical severity of the majority of patients in that box.

To investigate the correlation between the severity of XP-A phenotypes and the type and location of *XPA* germline variants, we identified 18 germline variants (9 novel) at the genomic DNA level (Figs 1B and 5). Our patient cohort carried *XPA* variants in exons 1, 3, 4, 5, 6, and introns 3 and 4. These variants included nonsense (n = 6), frameshift (n = 8), missense (n = 1), and splice (n = 3) variants. We identified 8 *XPA* variants associated with severe phenotypes, 6 variants associated with intermediate phenotypes, and 4 variants associated with mild phenotypes.

### Reduced post-UV survival and UDS in XP-A cells

**Fig 6:**
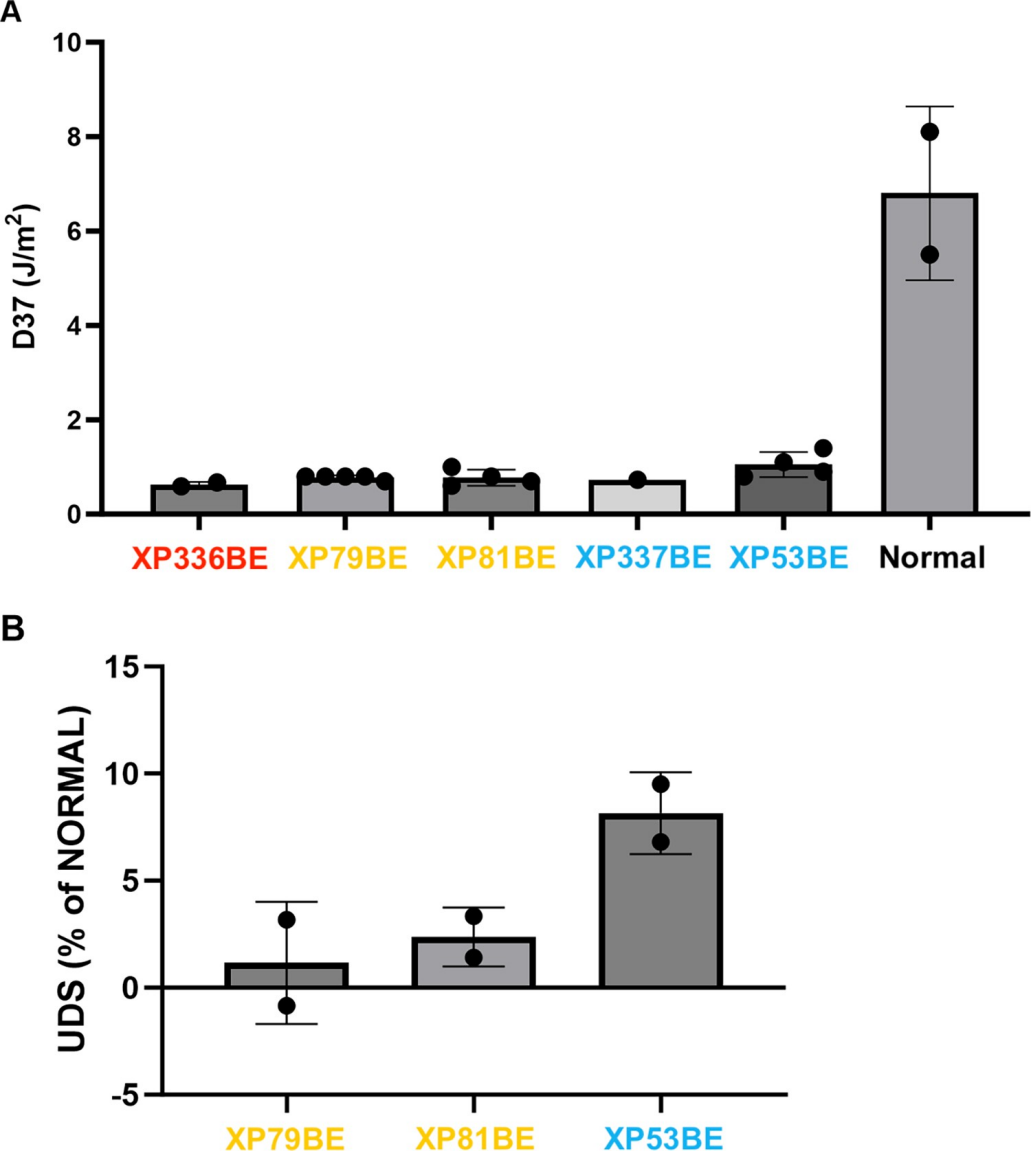
XP-A cells had reduced post-UV cell survival and UDS. (A) Cells from XP-A patients (XP336BE, XP79BE, XP81BE, XP337BE, and XP53BE) had reduced post-UV survival compared to normal cells. Bars indicate mean with SD. (B) The UDS from XP-A patients (XP79BE, XP81BE and XP53BE) was markedly reduced compared to that of normal donors. Bars indicate mean with SD. Colored XP cell lines indicate the clinical severity of the patient (see Fig 1).

The D_37_ (dose that results in 37% cell survival after UVC irradiation) was markedly reduced for the XP-A cells (XP336BE– 0.63 Jm^-2^, XP79BE– 0.78 Jm^-2^, XP81BE– 0.78 Jm^-2^, XP337BE– 0.73 Jm^-2^, and XP53BE– 1.05 Jm^-2^) in comparison to normal cells (5.5 to 8.1 Jm^-2^) (Fig 6A). The XP-A cells (XP79BE, XP81BE, and XP53BE) had markedly reduced UDS compared to normal cells (Fig 6B). These are characteristic features of cells from patients with *XPA* pathogenic variants [4,14,23–31].

### Severe XP-A phenotype

#### Clinical description of severe XP363BE patient

XP363BE was a 22-year-old Caucasian male diagnosed with XP at 18 months of age (Fig 1). He was the maternal uncle of severe patients XP360BE (nephew) [32] and XP631BE (niece). He sustained a severe blistering sunburn on his face and forearms after a 20-minute sun exposure at 6 weeks of age during the spring season in the Midwest region of the United States. He showed sun sensitivity and freckle-like pigmentation in his first year of life. He experienced delayed developmental milestones walking at 17 months. At age 7, he presented with persistent toe walking that progressively became less steady by age 9. These features were used to classify his neurological abnormality score as -2 at age 10 years (Figs 1 and 2 and Table 1). At age 12, he stopped walking and crawled until age 15. He then became non-ambulatory and required a G-tube due to dysphagia. At age 17, he developed mild to severe bilateral hearing loss. By age 20, he was profoundly deaf and could not speak [32]. He had consistent sun protection throughout his life and no skin cancers. He died at age 22 years due to progressive neurological complications [32].

#### Nonsense and frameshift variants causing premature stop codons in XPA exon 3 and exon 5 correlated with severe disease

All eight severe patients in our study (Fig 1) presented with gait disturbances due to spasticity or ataxia by age 10 years and carried nonsense or frameshift variants in exons 3 or 5 that resulted in premature stop codons. Similarly, Garcia-Moreno et al. (2023*)* categorized nonsense and frameshift variants in XPA exons 3 and 5 as the most severe, leading to complete protein inactivation [33]. In the literature, homozygotes with different missense, nonsense, and frameshift variants in exon 3 have reported to have severe cutaneous and neurological symptoms [15,26,31,34–36]. The p.Glu111* nonsense variant identified in XP120BE (Figs 1B and 5) was previously reported in three Tunisian siblings [36]. In patient cells with exon 3 variants, we found markedly reduced post-UV cell survival (D_37_: 0.63 Jm^-2^) (Fig 6A) and host cell reactivation (S2 Fig). Similarly, other laboratories reported markedly reduced DNA repair in cells with variants in exon 3. For instance, two Somalian patients (XP80BR and XP81BR) carried the p.Cys105Tyr missense variant and had markedly reduced UDS levels (2–5% of normal) [26]. A Japanese patient (XP18OS) carried the p.Try116* nonsense variant and showed significantly reduced post-UV survival (D_37_: 0.26 Jm^-2^) [34]. In addition, a Caucasian patient (XP1CA/GM02990), carrying the p.Thr125Ilefs*15 frameshift variant, showed significantly reduced post-UV colony forming ability [15,37,38]. In XP1CA, *XPA* mRNA was undetectable due to nonsense-mediated mRNA decay. This may explain the undetectable levels of XPA protein in XP120BE and XP360BE (S3 Fig).

We identified a novel p.Gln216* nonsense variant in exon 5 in hemizygotes XP40BE and XP509BE (Figs 1B and 5). In XP40BE, XPA protein was not detectable (S3 Fig). In the literature, severe cutaneous and neurological symptoms were also reported in other homozygotes carrying different frameshift and nonsense variants in exon 5 [15,26,36,39,40]. No missense variants have been reported in exon 5. In patient cells with exon 5 variants, previous laboratory studies revealed markedly reduced DNA repair. For instance, a Bangladeshi patient (XP57BR) carried the p.Met214Asnfs*7 frameshift variant and had exceptionally reduced UDS levels (1% of normal) [26]. A Palestinian patient (XP12RO) carried the p.Arg207* nonsense variant, showing markedly reduced post-UV colony forming ability [37]. Moreover, the *XPA* mRNA in XP12RO was detectable, but greatly reduced [40]. It appears that this exon 5 p.Arg207* nonsense variant may not trigger nonsense-mediated mRNA decay to the same extent as the exon 3 frameshift variant in XP1CA. The difference may be attributed to the C>T transition at position 619 of the XPA coding region in XP12RO.

Taken together, homozygous nonsense and frameshift premature stop variants in exons 3 and 5 appear to reduce the XPA protein to undetectable levels and can affect the functions of proteins that interact with XPA. This may disrupt essential protein-protein interactions that are important for efficient DNA repair (see below), which appears to contribute to severe disease.

### Intermediate XP-A phenotype

#### Clinical description of intermediate XP12BE patient

XP12BE was a 44-year-old Caucasian female diagnosed with XP at 4 years of age (Fig 1). She experienced early-onset sun-sensitivity, with her first acute reaction at age 3 months after minimal sun exposure during the spring season on the East Coast of the United States [41,42]. Her early developmental milestones were normal. Before the age of 4 years, she developed significant erythema, swelling, and freckle-like pigmentation on sun-exposed skin [41–43]. At age 7 years, she displayed early neurological abnormalities, such as areflexia and difficulties with tandem walking and rapid alternate movements. Psychometric testing revealed a Slosson full-scale IQ (FSIQ) score of 96 [41,42]. At age 8, she further presented with ataxia [42]. These features were used to classify her neurological abnormality score as -1 at age 10 years (Figs 1 and 2 and Table 1). Additionally, she had her first pathologically confirmed skin cancer (sclerosing basal cell carcinoma of the cheek) [41,42,44]. Thereafter, she developed many XP-related skin cancers throughout her life, including over 200 basal cell carcinomas and 1 squamous cell carcinoma [41–44]. By age 17, she developed mild bilateral hearing loss with a four-frequency pure tone average (4F-PTA) of 26.25 dbHL in both ears and was subsequently fitted with hearing aids [18]. Her Wechsler Intelligence Scale for Children-Revised Form (WISC-R) FSIQ was 76 [43]. By age 20, she was diagnosed with marked sensorineural hearing loss [18]. At age 21, nerve conduction studies showed sensory-motor peripheral neuropathy [19]. By age 24, she had cerebellar dysfunction involving gait. At age 36, she presented with mild dysphagia. At age 37, she relied on a walker for mobility assistance [41]. A G-tube was placed due to her inability to swallow food [25,44]. By age 41, she became non-ambulatory and had an FSIQ in the mid-60s. Her neurologic deterioration progressed, and she died by XP-related neurologic degeneration at age 44 years [41]. An autopsy was performed at the NIH that revealed generalized brain atrophy, resulting in marked dilation of the lateral ventricles, thinning of the corpus callosum, and a brain weight equivalent to that of a 6-month-old infant [25,41].

#### c.555G>C variant at last base in XPA exon 4 correlated with intermediate disease

We identified the c.555G>C variant in four patients with intermediate disease who had developmental delay (IQ of 50–85) by age 10 years (Figs 1B and 5). This caused a G>C transversion (ACA**G**/GT ➔ ACA**C**/GT) at the last nucleotide position in XPA exon 4, resulting in a missense variant of Gln185 to His. The severity of this variant has been suggested to likely have a major effect on protein function, but not cause the complete inactivation of the protein [33]. This agrees with our findings, suggesting that the c.555G>C variant is of intermediate severity. Splice donor sites are located in the 3’ end of the exon and 5’ end of the intron, consisting of AG/GT. The G>C transversion disrupted this canonical sequence in exon 4 and resulted in the elimination of the 5’ donor splice site in intron 4. This resulted in the activation of cryptic splice sites in exon 4 and intron 4, yielding 3 *XPA* mRNA isoforms (36bp in-frame insertion, 29bp deletion, and 6bp in-frame deletion) [45]. Laboratory findings on XP19BE (GM01630) cells had reduced amounts of normal-sized *XPA* mRNA [45]. This may explain why 4% of normal XPA protein was detected in homozygote XP19BE (S3 Fig). Additionally, XP19BE cells had markedly reduced post-UV colony-forming ability; however, this was slightly greater than that of XP2OS cells from a Japanese XP-A patient carrying the severe IVS3-1G>C splice founder variant [45].

Homozygotes with different nonsense and frameshift variants in exon 4 have been reported with skin lesions and neurological symptoms including developmental delay, spasticity, microcephaly, and moderate to severe hearing loss [46–48]. For instance, three Egyptian patients with intermediate disease (XP1GE, XP2UE, and XP7GI), ages 2–12 years, carried the homozygous p.Gln185* nonsense variant at the last base of XPA exon 4 [46,48]. Egyptian patient XP1GE exhibited spasticity by age 8 [48]. Patient XP7GI had microcephaly, cerebellar hypoplasia, hyperreflexia, and moderate to severe hearing loss by age 2 [46]. In contrast, our patient XP19BE experienced a delayed onset of symptoms, with moderate hearing loss emerging at age 24, followed by gait disturbance, axonal sensorimotor polyneuropathy, and dysphagia at age 33. The identified alternative splicing defect in XP19BE offers a potential explanation for the delayed onset of symptoms.

### Mild XP-A phenotype

#### Clinical description of mild XP53BE patient

XP53BE was a 40-year-old male of Indian ancestry (Fig 1). His parents were first cousins. He was born in the northwest region of India and experienced significant freckle-like pigmentation on his face at age 6 months. Subsequently, his parents were instructed to expose him to sunlight and apply oil to his face. This resulted in inconsolable crying and redness without blistering. He was the youngest of three children and was the only known case of XP in his family. He had a family history of diabetes and hypertension. His early developmental milestones were normal. He moved to Canada at age 5 years. He was diagnosed with XP at 8 years of age based on increased UV sensitivity and reduced UDS of his cultured fibroblasts (Fig 6A and 6B). These features were used to classify his neurological abnormality score as 0 at age 10 years (Figs 1 and 2 and Table 1). In high school, he was actively involved in sports, including wrestling and football. He graduated from a two-year junior college with a diploma in social work. At age 20, he was diagnosed with hypertension. He worked as a volunteer provincial constable for three years. At age 22, he developed his first skin cancer, a basal cell carcinoma (BCC) on his forehead. He underwent extensive testing at the NIH, which revealed normal hearing for speech and pure tones bilaterally, a normal nerve conduction study, a full-scale IQ of 78, and a normal MRI brain scan. Homozygous splicing variant of IVS4+8A>G (c.555+8A>G) in intron 4 was detected in the *XPA* DNA repair gene. At age 28, his hearing was normal. At age 30, he was diagnosed with type 2 diabetes. He married at age 35 and has no children. He used minimal sun protection and experienced non-blistering sunburns when outdoors. At age 37, he developed a second BCC on his forehead. He has worked in information technology since age 25. He self-reported no hearing loss, gait disturbances, or other neurological symptoms.

#### XPA intron 4 variant creates new splice donor site in mild XP53BE patient

In addition to XP53BE, we found a second mild patient, XP618BE (Figs 1 and 2), who carried the same homozygous IVS4+8A>G (c.555+8A>G) splice variant in intron 4 (Figs 1B and 5). This alternative splice variant generates a new splice donor site and is suggested to have a mild effect on protein function [33]. In addition, this is a founder variant in XP-A patients of Asian origins, particularly from Pakistan, India, and Afghanistan [26,31]. In XP53BE cells, this variant resulted in reduced post-UV survival (1.05 Jm^-2^) (Fig 6A) and UDS (8.2% of normal) (Fig 6B) compared to normal cells.

**Fig 7:**
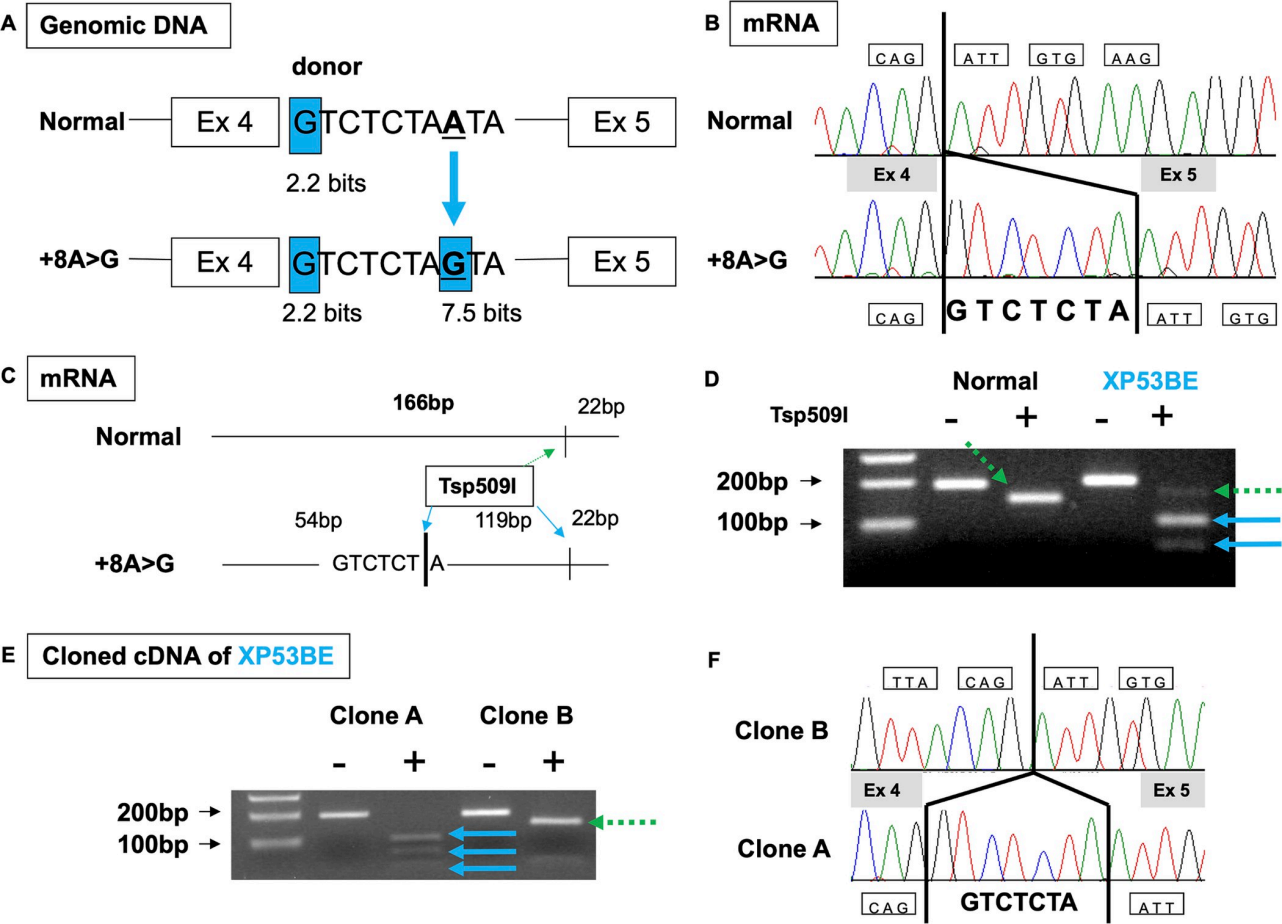
Normal splice isoform detected in mild XP53BE patient. (A) Sequencing analysis of genomic DNA showed an A to G change, which created a new strong donor site, eight bases downstream of the normal XPA exon 4 donor site. (B) Sequencing analysis of cDNA indicated a seven base GTCTCTA insertion between XPA exon 4 and exon 5. (C) Restriction fragment length polymorphism (RFLP). A new Tsp509I restriction cleavage site was created by the GTCTCTA insertion. (D) RFLP analysis. Amplified cDNA after Tsp509I digestion. XP53BE cDNA showed a normal band at 166bp and two abnormal bands at 119bp and 54bp. (E) XPA cDNA of XP53BE was subcloned and (F) sequenced. Dashed green arrows indicate normal message. Blue arrows indicate abnormal message.

Through genomic DNA sequencing, we identified an A to G single nucleotide change which created a new strong donor site (7.5 bits) eight bases downstream of the normal XPA exon 4 donor site (2.2 bits) (Fig 7A). We identified a *XPA* mRNA splice-isoform with 7-base insertion (GTCTCTA) (Fig 7B). This insertion utilized the new splice donor site created by the c.555+8A>G variant within the 188bp intron sequence located between XPA exons 4 and 5. The 7-base insertion created a new Tsp509I restriction cleavage site (Fig 7C and 7D). Upon Tsp509I digestion of the amplified cDNA, the normal sample showed a 166bp band. In contrast, XP53BE cDNA showed three bands. These included one normal band (166bp) and two abnormal bands (119bp and 54bp). The XP53BE cDNA was subcloned and sequenced, revealing two distinct clones (Fig 7E and 7F). Clone A showed the presence of the 7-base insertion. The normal sequence was detected in Clone B, confirming the absence of the 7-base insertion. This indicates the coexistence of both the new strong donor site (7.5 bits) and normal donor site (2.2 bits) in XP53BE. The normal donor site was not destroyed, resulting in the formation of some normal full-length *XPA* mRNA.

The clinical manifestations and functional tests of 14 other XP-A patients carrying the IVS4+8A>G (c.555+8A>G) alternative splice Pakistani/Indian founder variant in intron 4 are presented in S1 Table [26,31,49,50]. These patients had a late onset of skin cancers and displayed no neurological abnormalities or minimal neurological features, including mild developmental delay or absent deep tendon reflexes. These patients had a prolonged lifespan, with the oldest patient reported in the literature reaching 84 years of age [26,33]. Using our neurological assessment scoring scale, we were able to classify all 14 of the patients carrying this variant as having mild disease and a neurological abnormality score of 0 at age 10 years. Functional tests from different laboratories showed a range of 2–20% of normal UDS levels [26,31,50]. Additionally, these studies showed about 2–5% of normal splice *XPA* mRNA message and detected a small amount of normal XPA protein [31,50]. This suggests that a small amount of normal message is sufficient to exhibit mild disease. Similar observations were seen in XP-C patients where 3–5% of normal *XPC* mRNA led to a mild phenotype and partial protection against skin cancers [51,52].

#### Genomic DNA deletion results in exon 6 deletion in mild XP337BE patient

**Fig 8:**
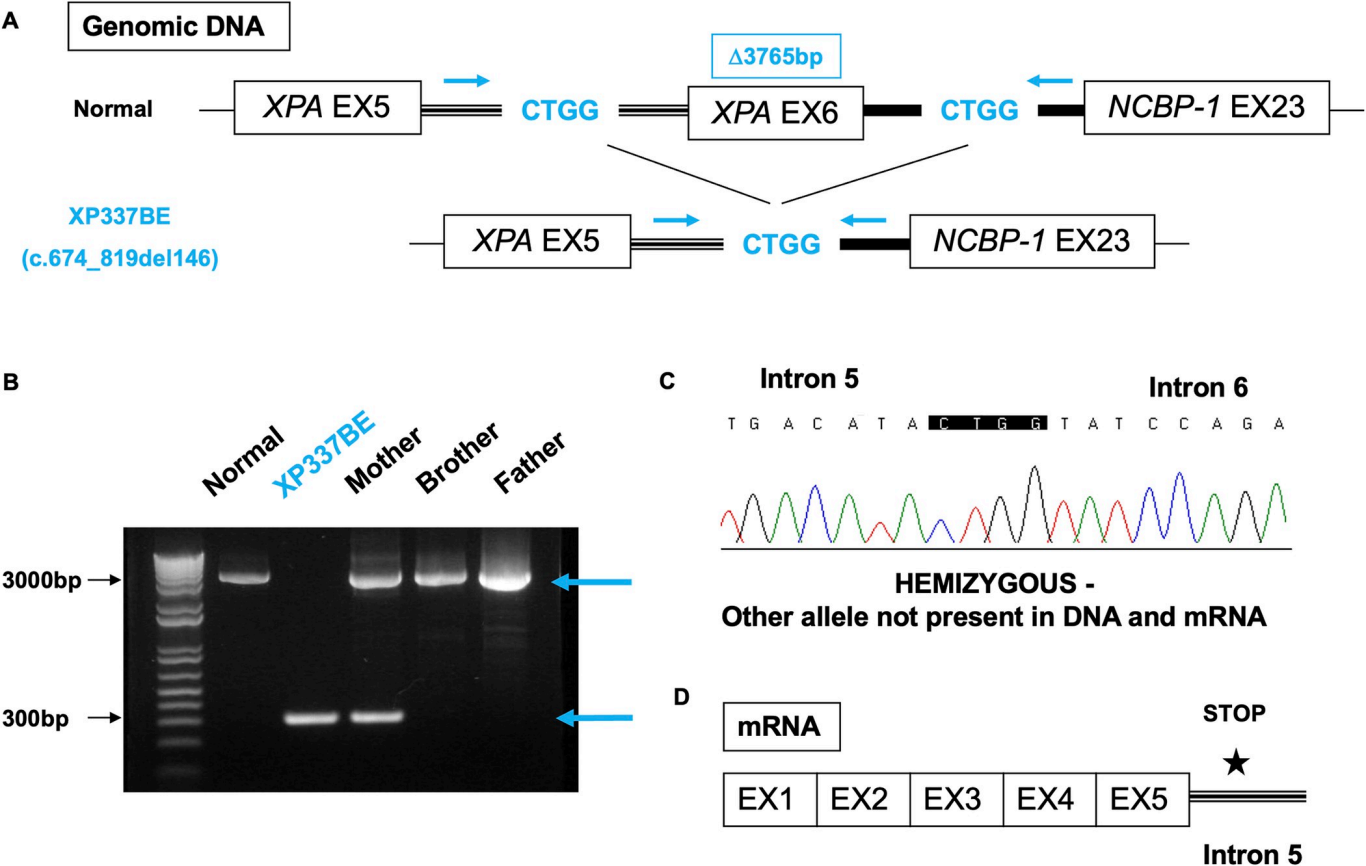
PCR and sequencing analysis detected a genomic DNA deletion that resulted in the complete deletion of XPA exon 6 in mild XP337BE patient. (A) Linear map of the amplification of XPA exon 5 and NCBP-1 exon 23 in XPA normal control. XP337BE showed an identical sequence of CTGG in introns 5 and 6. Horizontal lines represent introns. Black boxes represent exons. Arrows represent forward and reverse primers. (B) Agarose gel electrophoresis of genomic DNA PCR amplification products using XPA forward (intron 5) and *NCBP-1* reverse primer pair in XPA exon 6 normal control, XP337BE, her heterozygous mother, and her unaffected brother and father. Top blue arrow indicates normal size band (3765bp). Bottom blue arrow indicates short band (314bp). (C) Sequencing analysis of short band on the genomic DNA region between XPA intron 5 and intron 6. XP337BE was hemizygous as the other allele was not present in DNA and mRNA. (D) Linear map of XP337BE mRNA. Black boxes represent exons. Horizontal line represents intron 5. Star (★) represents frameshift deletion that causes a premature stop codon.

XP337BE was hemizygous for c.674_819del (g.20108_23558del; p.E225Vfs*4) and displayed mild clinical features (Figs 1A, 1B, 2, 5 and 8). We investigated the neighboring gene *NCBP-1*, which is located downstream of *XPA*. We successfully amplified NCBP-1 exon 23 in both XP337BE and normal and found an identical CTGG sequence in XPA introns 5 and 6 (Fig 8A). We designed a pair of primers, with the forward primer located in XPA intron 5 and the reverse primer in NCBP-1 intron 6, to amplify a 3765bp region on the genomic DNA. The DNA from the mother, father, and unaffected brother showed a 3765bp product. The DNA from the heterozygous mother showed both a 3765bp product and a shorter 314bp product (Fig 8B). Only the shorter 314bp product was detected in XP337BE. Sequencing of this shorter product revealed a 3451bp deletion between XPA intron 5 and intron 6 (Fig 8C), resulting in the complete deletion of XPA exon 6 (Fig 8D). These findings indicate that the deletion of XPA exon 6 is associated with a mild clinical phenotype.

Similar laboratory findings were observed in four mild Japanese XP-A compound heterozygotes (XP17HM, XP21HM, XP42HM, and XP43HM), each carrying an exon 6 variant on one allele, compared to a severe Japanese XP-A homozygote carrying the splice founder variant (D_37_: 0.8–1.4 Jm^-2^ versus 0.4 Jm^-2^) [13]. Mild Japanese XP-A patients XP17HM and XP21HM were of a similar age to our mild patient XP337BE (age 31), had mild developmental delay (FSIQ of 72), and no skin cancers at last observation. XP17HM and XP21HM carried an insertion variant in exon 6 and had detectable truncated XPA protein [13]. In contrast, XP337BE had a large C-terminal deletion that deleted exon 6 (Figs 1B and 8D), and no normal-sized XPA protein was detected (S3 Fig).

### Compound heterozygotes

#### Re-initiation in XPA exon 2 in mild XP315BE patient

We investigated the impact of compound heterozygosity on XP-A phenotypes. Reports indicated that carrying two alleles with *XPA* pathogenic variants within exon 3 and exon 5, as well as intron 3, results in a severe phenotype [12,36,53]. In accordance, we described five severe compound heterozygotes XP360BE (and relatives XP363BE and XP631BE) (c.288delT, p.Val97Leufs*7; c.349_353delCTTAT, p.Leu117Glufs*4), XP558BE (c.315C>A, p.Cys105*; c.324T>A, p.Cys108*), and XP336BE (c.338_339delTG, p.Met113Argfs*9; c.603_607delAGAAG, p.Glu201Aspfs*18) who carried a variant in exon 3 and a second variant in exon 5 (Figs 1B and 5). These severe patients presented with early-onset neurological symptoms, including developmental delay (IQ<50), gait disturbance due to spasticity or ataxia, an inability to walk, peripheral neuropathy, dysphagia at age 10 and early death (Figs 1, 2 and S1). Both alleles in patient XP360BE had reduced HCR DNA repair activity (S2 Fig).

On the other hand, when the second variant allele was outside exon 3, intron 3, or exon 5, patients had milder symptoms [13]. Unlike the severe XP-A patients, mild patient XP315BE was alive at age 35 years. She had normal developmental milestones, graduated from high school, and lived independently. She had minimal neurological abnormalities, consisting only of absent deep tendon reflexes with a normal gait, and normal hearing. She developed a seizure disorder beginning at age 33 but had a normal MRI of the brain. Mild patient XP315BE carried the same p.Leu117Glufs*4 frameshift variant in exon 3 as severe patients XP360BE, XP363BE, and XP631BE. She carried a novel c.142delT (p.Tyr48Thrfs*15) variant on the other allele in XPA exon 1 (Figs 1B and 5).

**Fig 9:**
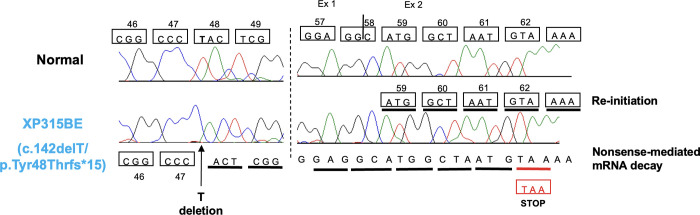
Re-initiation in mild XP315BE XP-A patient. Sequencing analysis of genomic DNA revealed a T deletion at position 142, which produced a frameshift variant in exon 1, and resulted in a premature stop codon in exon 2 and possible nonsense-mediated mRNA decay. Re-initiation of RNA from the second methionine (ATG sequence) at codon 59 was predicted to occur in exon 2.

The c.142delT (p.Tyr48Thrfs*15) variant in *XPA* gene identified in mild patient XP315BE creates an early termination. The deletion of T changes Tyr 48 to Thr, which caused a frameshift with 14 new amino acids and a termination codon (TAA) in exon 2 of the *XPA* gene. This may be a candidate for nonsense-mediated decay. As a consequence of the termination of translation at the uORF stop codon due to deletion of T, the ribosomes reinitiate the translation at a downstream AUG, most probably in exon 2 (59ATG) or AUG in other exons of the *XPA* gene (Fig 9). The sequences at the natural Transcription Initiation Site (TIS) site were CAGAG**ATG**GCGGC and the sequence around 59ATG in exon 2 was GAGGC**ATG**GCTAA. The ATG in exon 2 appears to be a strong translation start site that can be utilized to start the re-initiation if the natural TIS is not available. The HCR assay experiment with an expression vector containing this mutant found increased DNA repair levels in the cells, which may be due to re-initiation of translation at the next downstream AUGs, most probably AUG in exon 2 (S2 Fig). We lack experimental data to predict the re-initiation at a specific downstream start codon. This suggests that the re-initiation contributes to the mild clinical phenotype in patient XP315BE [54–61]. Further, both alleles contribute to the clinical phenotype.

#### p.R228* nonsense variant in XPA exon 6 correlated with intermediate and mild disease

We identified the p.R228* nonsense variant in *XPA* exon 6 in compound heterozygote XP591BE who had intermediate disease features (Figs 1B and 5). Garcia-Moreno et al. (2023) also categorized this nonsense variant as a truncating variant near the C-terminal, suggesting some possible activity and likely to have an intermediate effect on protein function [33]. This variant is a prevalent founder variant observed in North African patients [30,62]. In the literature, 35 homozygous patients were described to have a range of progressive neurodegeneration (S2 Table). Using our neurological assessment scoring scale (Table 1), 28 cases (ages 7–34 years) were classified as intermediate disease [26,28–31,34,62–66]. Surprisingly, 7 cases (ages 17–35 years) were classified as mild disease [30,63,66]. These phenotypic differences may be due to the effects of unknown modifier genes. Functional tests among the homozygous patients revealed 1–8% of normal UDS [30,67] and markedly reduced post-UV cell survival (D_37_ of 0.55–0.80 Jm^-2^; LD_50_ of 5 Jm^-2^) [29,30,34]. Establishing a clear correlation between clinically intermediate and mild diseases and post-UV survival, UDS, or protein expression tests for these homozygotes was not possible due to the insufficient clinical features reported [26,28–31,34].

#### p.R258Yfs*5 frameshift variant in exon 6 in mild XP607BE patient

Intermediate patients XP79BE (p.C126W, p.Gln185His) and XP12BE (c.390-1G>T, p.Gln185His) each carried a second allele with a c.555G>C splice variant at the last base in the DNA-binding domain in exon 4 (Figs 1B and 5). Clinical manifestations in XP79BE and XP12BE included developmental delay (IQ of 50–85) by age 10 years and the onset of gait disturbance between ages 24–33. These patient cells had reduced UDS levels, ranging from 0–2% of normal (Fig 6B). In XP12BE, XPA protein was detected at 31% of normal levels (S3 Fig) [45].

However, mild patient XP607BE carried the same c.555G>C (p.Gln185His) variant as intermediate patients XP79BE and XP12BE. On the second allele, XP607BE carried the p.Arg258Tyrfs*5 frameshift variant in exon 6 (Figs 1B and 5). In contrast to severe and intermediate patients, XP607BE had a delayed onset of symptoms. Neurological manifestations began with developmental delay (IQ 50–85) at age 18 years, followed by gait disturbance at age 37. By age 39, brain atrophy, peripheral sensorimotor neuropathy, and dementia had developed. At age 45, XP607BE was the oldest XP-A patient in our study. He relied on a walker for mobility assistance. This suggests that the mild phenotype may be attributed to the exon 6 variants, where one XPA allele predominates over the other allele.

This p.Arg258Tyrfs*5 frameshift variant in exon 6 was previously identified in five Hungarian XP-A compound heterozygote siblings [68]. The Hungarian siblings (ages 25–42) had an onset of neurological symptoms at ages 13, mid-20s, or early-30s, with clinical manifestations similar to XP607BE. However, one sibling died at age 39. Neuropathological findings revealed pronounced generalized atrophy, thinning of the cortical ribbon and cerebellar folia, and a total brain weight equivalent to that of a 7-month-old infant [68,69]. This was similar to neuropathological findings observed in intermediate patient XP12BE, who died at age 44 and had a brain weight equivalent to that of a 6-month-old infant [41].

Taken together, it appears that variants on both alleles contribute to phenotypic differences. Similar observations were seen in XP-D patients where both XPD alleles contributed to the phenotype of compound heterozygote XP patients [70].

### Relation of protein structure to clinical phenotype

**Fig 10:**
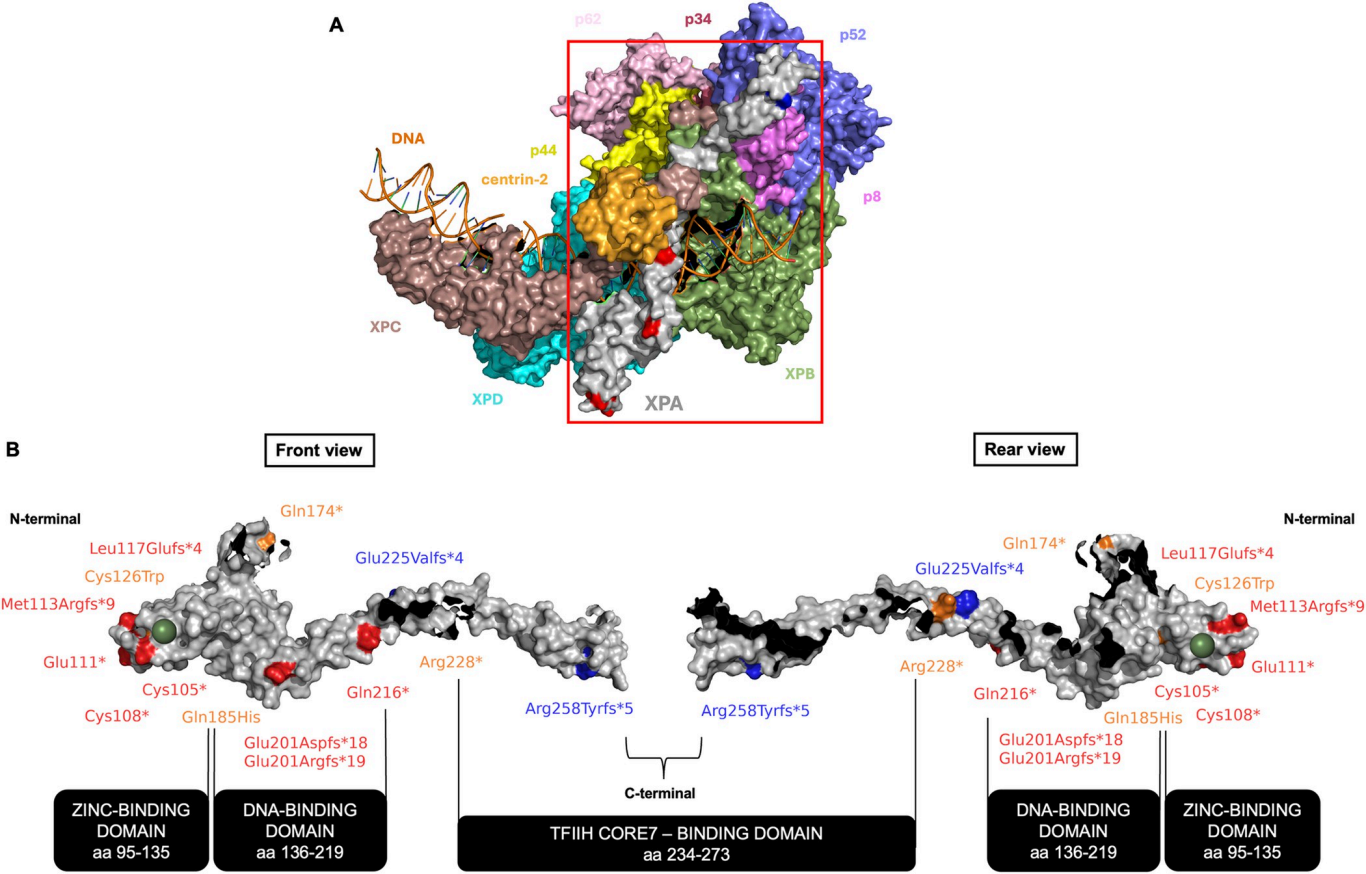
Mapping of *XPA* germline variants identified in NIH XP-A patients on XPA protein structure. (A) Surface structure of XPA repositioning Core7 of TFIIH relative to XPC-DNA lesion (PDB: 8EBT) [8,79]. TFIIH Core7 subunits XPB, XPD, p62, p52, p44, p34, and p8 are colored in green, cyan, light pink, slate, yellow, raspberry, and violet, respectively. XPC (brown), XPA (gray), centrin-2 (light orange), and DNA (dark orange) are shown. (B) Front and rear view of surface structure of XPA protein (amino acids 98–273) (PDB: 8EBT) [8,79]. N-terminal is located at the left of the structure (front view). C-terminal is located at the right of the structure (front view). Severe (red) variants are clustered in exons 3 and 5. Exon 3 encodes the zinc-finger binding domain (green sphere) for RPA interactions. Intermediate (gold) variants are clustered within the DNA-binding domain of exon 4. Mild (blue) variants are clustered within exons 1 and 6. Exon 1 interacts with RPA. Exon 2 interacts with ERCC1-XPF. Exon 6 interacts with TFIIH Core7. Black regions show the protein interaction sites from Fig 10A.

To determine the spatial distribution of variants on the XPA protein, we mapped the pathogenic variants characterized in this study onto the XPA protein structure (Fig 10 and S1 and S2 Videos) (see methods for details). XPA contains three structural domains, a central folded DNA-binding domain (aa 98–239) flanked by disordered N-terminal (aa 1–97) and C-terminal (aa 240–273) domains that mediate interactions with other NER factors. The cryoEM structure of human XPA has been determined from amino acids 98 to 273 and was visualized by PyMOL [8,71,72].

Nonsense and frameshift premature stop variants, localized in *XPA* exon 3 (aa 95–129) and exon 5 (aa 186–224), correlated with clinically severe disease. Exon 3 pathogenic variants may disrupt the interaction between XPA and RPA via the zinc-finger binding motif. This disruption may lead to inefficient or incomplete DNA repair [73–78]. This interaction is important for positioning XPA close to the DNA substrate, thereby licensing the full complex assembly and 5’ incision by ERCC1-XPF endonuclease during nucleotide excision repair [76].

In exons 4–5 (aa 130–224), amino acids 136–219 are mainly involved with DNA-binding, but are also involved in protein interaction with XPB, RPA, XPC, and XPD [8,75,76,78–83]. Exon 5 variants (aa 186–224), localized in the DNA-binding domain (aa 130–224), may disrupt the interaction of XPA protein with DDB2 (XPE), which is required to establish a stable XPC-TFIIH-DNA complex (Fig 10A) [84].

The c.555G>C pathogenic variant, localized in the last base of exon 4 (aa 130–185), correlated with clinically intermediate disease. This change results in a missense variant (p.Gln185His) and alternative splicing that yielded three *XPA* mRNA isoforms (36 bp in-frame insertion, 29 bp deletion, and 6 bp in-frame deletion) [45] (Figs 1B and 5).

Pathogenic variants localized near the N-terminal disordered domain of XPA (Fig 1B) correlated with clinically mild disease (Figs 1B and 5). These XPA variants may disrupt the N-terminal disordered domain, which has been reported to interact with RPA [76].

Patients also had mild disease associated with variants in the C-terminal region in exon 6 (aa 225–273), which is known to interact with TFIIH Core7 [8,17]. XPA repositions TFIIH Core7 relative to XPC-lesion DNA, causing the lesion to be located downstream and 3’ to XPD. Once XPA dissociates from the CAK module of TFIIH, Core7 binds to the lesion DNA as if it were recruited by XPC. This sequence of events enables subsequent nucleotide excision repair reactions, following the pathways of both global genome repair (GG-NER) and transcription-coupled repair (TC-NER) [8]. A recent study suggests that C-terminal disease-associated *XPA* variants can impair interaction with TFIIH that affect TC-NER to a greater extent than GG-NER [17]. The mild phenotype may be a result of slightly increased post-UV cell survival (Fig 6A). Interestingly, the observation that the deletion of XPA exon 6 is associated with a mild clinical phenotype suggests that XPA protein encoded from exons 1–5 have sufficient function or functional domains to prevent severe neurological abnormalities.

Taken together, this data suggests that the localization of XPA pathogenic variants within specific XPA regions, along with the protein-DNA and protein-protein interactions in those regions, play a role in contributing to the severity of the observed clinical phenotypes.

### Limitations

Our study has several limitations. Since XP is an extremely rare disease, the sample size of our XP-A patient cohort was small, thus the severity classification was based on a small number of patients and laboratory tests such as UDS, post-UV cell survival, and HCR. However, its multi-decade duration provides insights into long-term effects on patient morbidity and mortality. Large patient cohorts would help identify additional clinical features and germline variants that were not captured in the current study. The values of the scores within the neurological assessment scale have some degree of clinical subjectivity. Future studies should focus on refining and standardizing the values of the scoring system. A more detailed scoring system [33] has recently been proposed that may be more appropriate for in-depth clinical follow-up of XP-A patients. Additionally, the performance of post-UV survival, host cell reactivation, and UDS tests may be influenced by unknown factors in different laboratories.

## Materials and methods

### Cell lines, culture conditions, and DNA/RNA isolation

Normal primary skin fibroblasts (AG13145) and lymphoblastoid cell (KR06057) and XP-A cells from patients (XP12BE–GM05509 and GM02250, XP19BE–GM01630, XP79BE, XP81BE, XP315BE, XP360BE, XP363BE, XP120BE–GM16616 and GM16615, XP336BE, XP337BE, XP40BE–GM13295, XP53BE), the mother of XP12BE (XPH250BE–GM05510 and GM05511), father of XP12BE (XPH251BE–GM05568 and GM05569), mother of XP120BE (XPH121BE–GM16614 and GM16613), and mother of XP315BE (XPH316BE) and sister of XP360BE (XP631BE) and normal primary skin fibroblasts (AG13145) and lymphoblastoid cell were obtained from the Human Genetic Mutant Cell Repository (Camden, NJ). Fibroblast cell lines were grown in Dulbecco’s modified Eagle’s medium (DMEM) (Invitrogen) containing 40mM glutamine and 10% fetal bovine serum (Gibco). Lymphoblast cells were grown in RPMI 1640 medium with 15% fetal bovine serum as described previously [24]. DNA was isolated utilizing DNAzol reagent as per the vendor’s protocol (Invitrogen). Total cytoplasmic RNA was isolated from cells by the RNAqueous small scale phenol-free total RNA isolation kit (Ambion, Austin, TX) according to the vendor’s protocol.

### DNA repair measurement and complementation group assignment

Cell survival was measured by assessing cell growth in microwell plates following exposure to UVC dose of 2–16 Jm^-2^ [85,86]. DNA repair abnormalities were assessed as reduced post-UV unscheduled DNA synthesis (UDS) [1]. The UDS level was measured as the average number of grains per nucleus as a function of UV dose to cells. In the early years of this study, the pCMVLuc reporter gene plasmid (a generous gift from M. Hedayati and L. Grossman, Johns Hopkins University, Baltimore, MD) was used to assign some XP cells to a specific complementation group as described previously [23]. Briefly, 4 uL (200ng) of CsCl-purified pCMVLuc, either UVC-irradiated (1000 J/m^2^) or unirradiated, was transfected into fibroblasts and lymphoblastoid cells using 4 uL lipofectamine (Invitrogen), co-transfection with a panel of wild-type cDNAs encoding the DNA repair genes XPA to XPG [87]. After 48 hours, the luciferase activity was measured with a TD20/20 luminometer (Monolight 2010; Analytical Luminescence Laboratory, San Diego, CA) using luciferase assay reagent (Promega) as per the vendor’s protocol. Relative luciferase activities are shown as a percentage of activities obtained with UV-irradiated versus unirradiated control plasmid (S4 Fig). Only co-transfection with a plasmid containing the normal XPA cDNA led to a markedly increased post-UV HCR, thereby assigning the tested cells (XP337BE) to XP complementation group A. All complementation group assignments were confirmed by DNA sequencing. In subsequent years, DNA sequencing only was used for screening (see below).

### Host cell reactivation assay for DNA repair levels in cells

The DNA repair levels in the fibroblast cells was measured employing post-UV host cell reactivation assay using the pCMVLuc reporter gene plasmid as described [23]. In brief, 4 μl (200 ng) pCMVLuc, either UV-irradiated (1000 J/m^2^) or unirradiated, was co-transfected with either a plasmid containing wild-type XPA cDNAs encoding the normal message or a plasmid containing mutant XPA cDNA encoding mutant message into cells using Lipofectamine (Invitrogen). After 48 h, the luciferase activity was measured with a luminometer (Monolight 2010; Analytical Luminescence Laboratory, San Diego, CA) using luciferase assay reagent (Promega) as per the vendor’s protocol. Relative luciferase activities are presented as a percentage of activities obtained with UV-irradiated versus unirradiated control plasmids.

### Western blotting

Proteins from cellular extracts were analyzed by SDS-polyacrylamide gel electrophoresis using 10% gels. For the Western blot analysis, 50 ug of total protein of cell extracts per lane were transferred to Hybond-C membrane (Amersham Pharmacia Biotech) and probed with the specific antibodies: anti-XPA polyclonal (Santa Cruz Biotechnology, Santa Cruz, CA) and anti-Actin polyclonal (Santa Cruz). The secondary anti-rabbit peroxidase-conjugate immunoglobulin G (IgG-HRP) was obtained from Santa Cruz. Proteins were detected using ECL Western blotting detection reagents (Amersham) as described previously [24].

### PCR amplification and nucleotide sequence analysis

Genomic DNA was isolated from fresh blood samples of XP-A patients and cell lines (fibroblasts or lymphoblasts) established from the XP-A patients, as described previously [23,24,88]. For sequencing, the six XPA exons were amplified with a series of pairs of primers (I-11/12, II-296/297, III-79/139, IV-41/42, V-126/52, VI-61/62) [15,37]. A pair of primers (Forward XPA-# TCATGTCACCGTGGCTAGCTAA, Reverse XPA-# CTTGCCAACCTATGTAGAGCAGG) were used for amplifying 3765bp part of XPA between intron 5 and intron 6. NCBP-1 exon 23 was amplified using a set of primers (Forward NCBP-# CCACCAGTTTTAGAGTAGAGTG, Reverse NCBP-# TTTTCCCCTTCCTCTTTTCATC). Two micrograms total RNA were reverse transcribed using the Superscript first strand synthesis system and oligo (dT)12-18 primers for first strand cDNA synthesis according to the manufacturer’s protocol (Invitrogen). The entire coding region of XPA was then amplified with primers (RT-E1F and RT-E6R: [89]) using the Advantage GC genomic PCR Kit (BD Bioscience). After agarose gel purification, the full length XPA cDNA was then subcloned into pCR2.1-TOPO vector (TOPO TA cloning kit, Invitrogen) and sequenced. PCR products were sequenced using the Thermo Sequenase II dye-terminator cycle sequencing Premix Kit (Amersham Pharmacia). Sequencing was performed by cycle sequencing employing dideoxy termination chemistry and an ABI 373A automated DNA sequencer (P.E. Applied Biosystems) using appropriate primers as described previously [23,90].

### Restriction fragment length polymorphism (RFLP) assay

The amplified DNA was digested with the appropriate enzyme. The genetic changes were analyzed by Eco0109I, BsaB1, or MboII restriction endonuclease (New England Biolabs). The fragments were resolved on agarose gel as described previously [88].

### Human Gene Mutation Database (HGMD)

We searched the Human Gene Mutation Database (HGMD) (professional version, 2024.2 release) [91,92] to identify *XPA* germline variants found in our NIH cohort. Variants not found in HGMD were classified as novel. Out of the 519,879 variants in the HGMD database, 72 were identified as *XPA* variants. The variants were categorized by HGMD as pathogenic, conflicting interpretations of pathogenicity, or not listed in the database.

### Protein structural analysis

The surface representation of XPA protein repositioning Core7 of TFIIH relative to XPC-DNA lesion (PDB: 8EBT) was generated using PyMOL Molecular Graphics System (Version 3.0.4 Schrödinger, LLC.). Germline variants in the *XPA* gene identified in the NIH cohort were then mapped onto the XPA protein of the 8EBT surface structure. On PyMOL, the variants were color-coded (mild = blue, intermediate = gold, severe = red) based on the observed clinical severity of the patients [8,73,79].

### Statistical analysis

Statistical analyses were performed using GraphPad Prism version 9.5.1 (GraphPad Software, San Diego, California, USA) or Microsoft Excel. Kaplan-Meier analyses were used to determine the age of onset of different events. Kaplan-Meier median age of onset and 95% confidence intervals were shown. Group comparisons were evaluated using the log-rank (Mantel-Cox) test. Statistical significance was defined as p < 0.05. The mean ± SEM values were used to present the data (see Fig legends).

## Supporting information

S1 Fig Unsupervised analysis of neurological degeneration in NIH XP-A patients. Neurological abnormality severity scores (Table 1) are plotted against patients’ age. Solid colored lines represent severe (red), intermediate (gold), and mild (blue) NIH XP-A patients. Black dotted line represents age 10 years. Open symbols indicate living patients. Closed symbols indicate deceased patients. Dotted colored lines represent, severe [12], intermediate [93,94], and mild [93,94] XP-A Japanese patients reported previously. Y-axis was displaced for clarity in patient data set. (A) Developmental delay in 18 patients. (B) Gait disturbance in 18 patients. (C) Peripheral neuropathy in 13 patients. (D) Hearing loss in 18 patients. (E) Dysphagia in 11 patients. These are the individual scores of the same patients that were summarized in Fig 3 [19,32,41,42].
(TIF)

S2 Fig Host cell reactivation (HCR) assay of mutated XPA plasmids. HCR assay was performed via co-transfection of the UV transfected reporter gene plasmid (pLUC) with XPA complementary DNA containing mutated plasmids from mild (blue), intermediate (gold), and severe (red) XP-A patients into fibroblasts from XP2OS (see methods section for details). (A) Graph. (B) Data.
(TIF)

S3 Fig XPA protein levels in cells from 10 NIH XP-A patients and normal control. Western blotting was performed on extracts from fibroblasts and lymphoblasts probed with antibody for XPA (upper) and ß-actin (lower) in normal control, (A) XP336BE, XP360BE, XP315BE, XP81BE, XP337BE, XP12BE, (B) XP40BE, (C) XP120BE, XP19BE, and XP53BE. XPA protein indicated with arrows. XPA protein was reduced in XP12BE (31% of normal), XP19BE (4% of normal), and XP53BE (6% of normal). No detectable XPA protein in other XP-A patients (see methods section for details).
(TIF)

S4 Fig XP cells were assigned to XP-A by host cell reactivation assay. UV transfected reporter gene plasmid (pLUC) was co-transfected with XP complementary DNA containing plasmid [pXPA, pXPC, pXPD, and pXPG] into fibroblasts from XP337BE (see methods section for details). The correction was achieved only by co-transfection of pXPA indicating that these cells are in XP-A (see methods section for details). (A) Graph. (B) Data.
(TIF)

S1 Table Summary of XPA c.555+8A>G splicing founder variant in 16 Pakistani and Indian XP-A patients. ^a^Neurological severity was assessed using our neurological abnormality scoring scale in Table 1. “Unknown” indicates patients under age 10 years or with insufficient information to classify. ^b^Number of patients classified. ^c^Age of patients classified. **UDS**: unscheduled DNA synthesis **D**_**37**_: dose that results in 37% cell survival after UVC irradiation
(DOCX)

S2 Table Summary of XPA p.R228* nonsense founder variant in 49 North African XP-A patients. ^a^Neurological severity was assessed using our neurological abnormality scoring scale in Table 1. “Unknown” indicates patients under age 10 years or with insufficient information to classify. ^b^Number of patients classified. ^c^Age of patients classified. ^d^One intermediate patient died at age 35 years from neurological degeneration and multiple organ failure. ^e^One intermediate patient died at age 32 years from pneumonia ^f^Ref [27]. **UDS**: unscheduled DNA synthesis **D**_**37**_: dose that results in 37% cell survival after UVC irradiation **LD**_**50**_: median lethal dose **RRS**: recovery of RNA synthesis after DNA damage
(DOCX)

S1 Video Surface structure of the XPA repositioning Core7 of TFIIH relative to XPC-DNA lesion (PDB: 8EBT) [8,79]. TFIIH Core7 subunits XPB, XPD, p62, p52, p44, p34, and p8 are colored in green, cyan, light pink, slate, yellow, raspberry, and violet, respectively. XPC (brown), XPA (gray), centrin-2 (light orange), and DNA (dark orange) are shown. See Fig 10A for details.
(MPG)

S2 Video XPA protein structure mapped with variants in NIH XP-A patients (PDB: 8EBT) [8,79]. XPA amino acids 98–273 are presented. Severe (red), intermediate (gold), and mild (blue) variants are shown. See Fig 10B for details.
(MPG)
