# Supplementary figures and images for: Different germline variants in the XPA gene are associated with severe, intermediate, or mild neurodegeneration in xeroderma pigmentosum patients
Source: PLoS Genet. 2024 Dec 2;20(12):e1011265. doi: 10.1371/journal.pgen.1011265 (PMC11637439; doi:10.1371/journal.pgen.1011265)

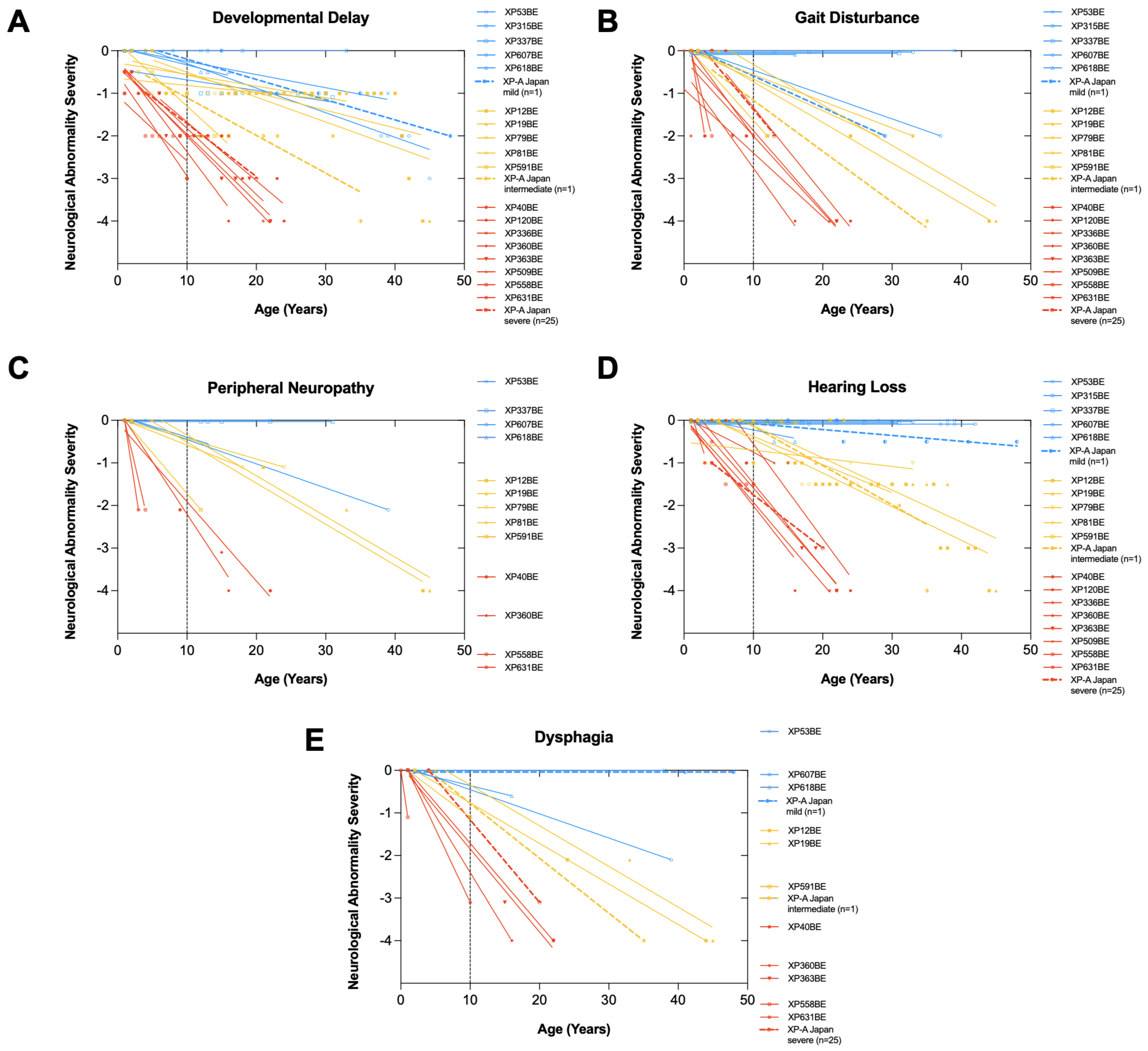

Supplement: S1 Fig — Neurological abnormality severity scores (Table 1) are plotted against patients’ age. Solid colored lines represent severe (red), intermediate (gold), and mild (blue) NIH XP-A patients. Black dotted line represents age 10 years. Open symbols indicate living patients. Closed symbols indicate deceased patients. Dotted colored lines represent, severe [12], intermediate [93,94], and mild [93,94] XP-A Japanese patients reported previously. Y-axis was displaced for clarity in patient data set. (A) Developmental delay in 18 patients. (B) Gait disturbance in 18 patients. (C) Peripheral neuropathy in 13 patients. (D) Hearing loss in 18 patients. (E) Dysphagia in 11 patients. These are the individual scores of the same patients that were summarized in Fig 3 [19,32,41,42]. (TIF) [file pgen.1011265.s001.tif]

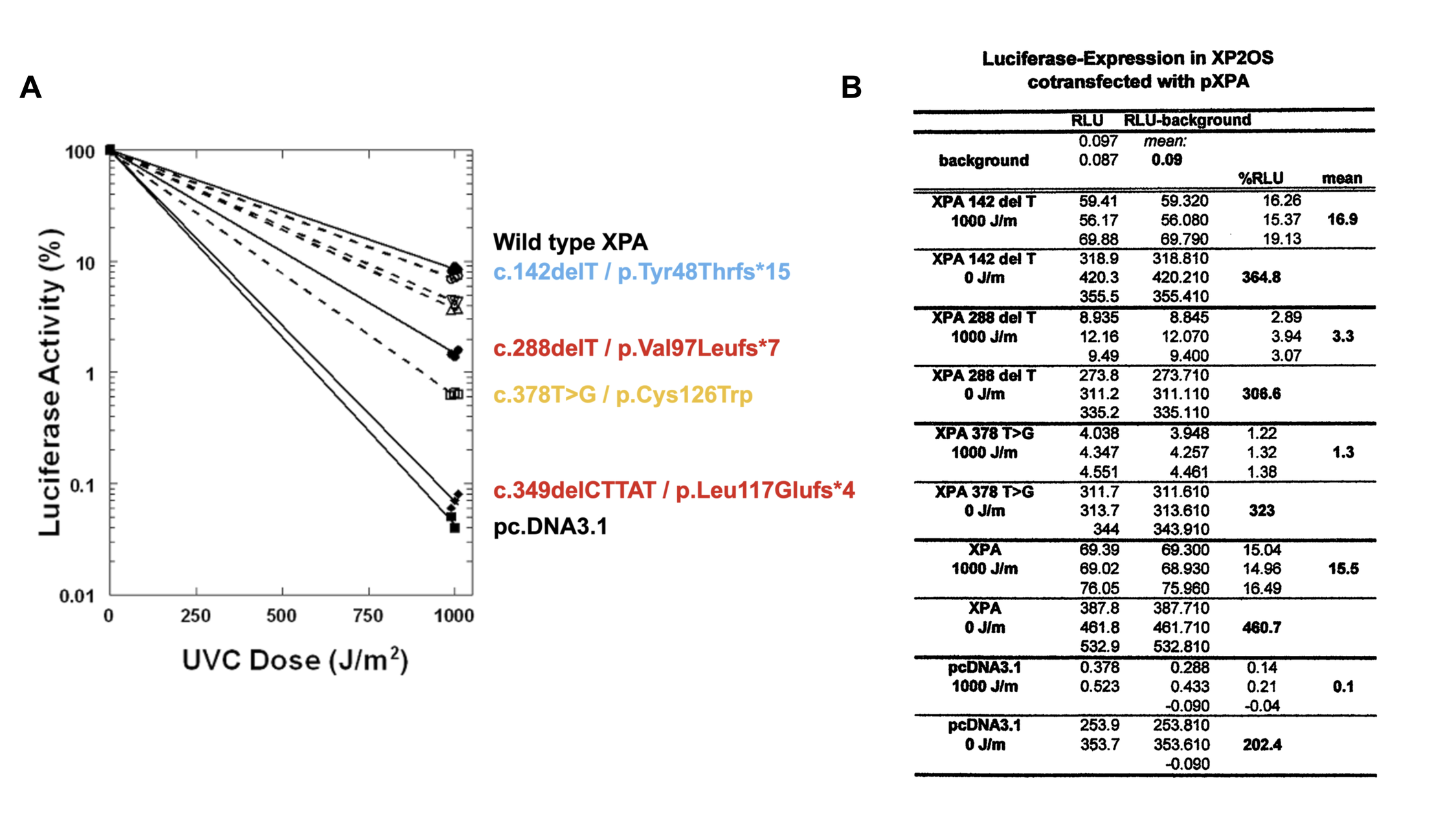

Supplement: S2 Fig — HCR assay was performed via co-transfection of the UV transfected reporter gene plasmid (pLUC) with XPA complementary DNA containing mutated plasmids from mild (blue), intermediate (gold), and severe (red) XP-A patients into fibroblasts from XP2OS (see methods section for details). (A) Graph. (B) Data. (TIF) [file pgen.1011265.s002.tif]

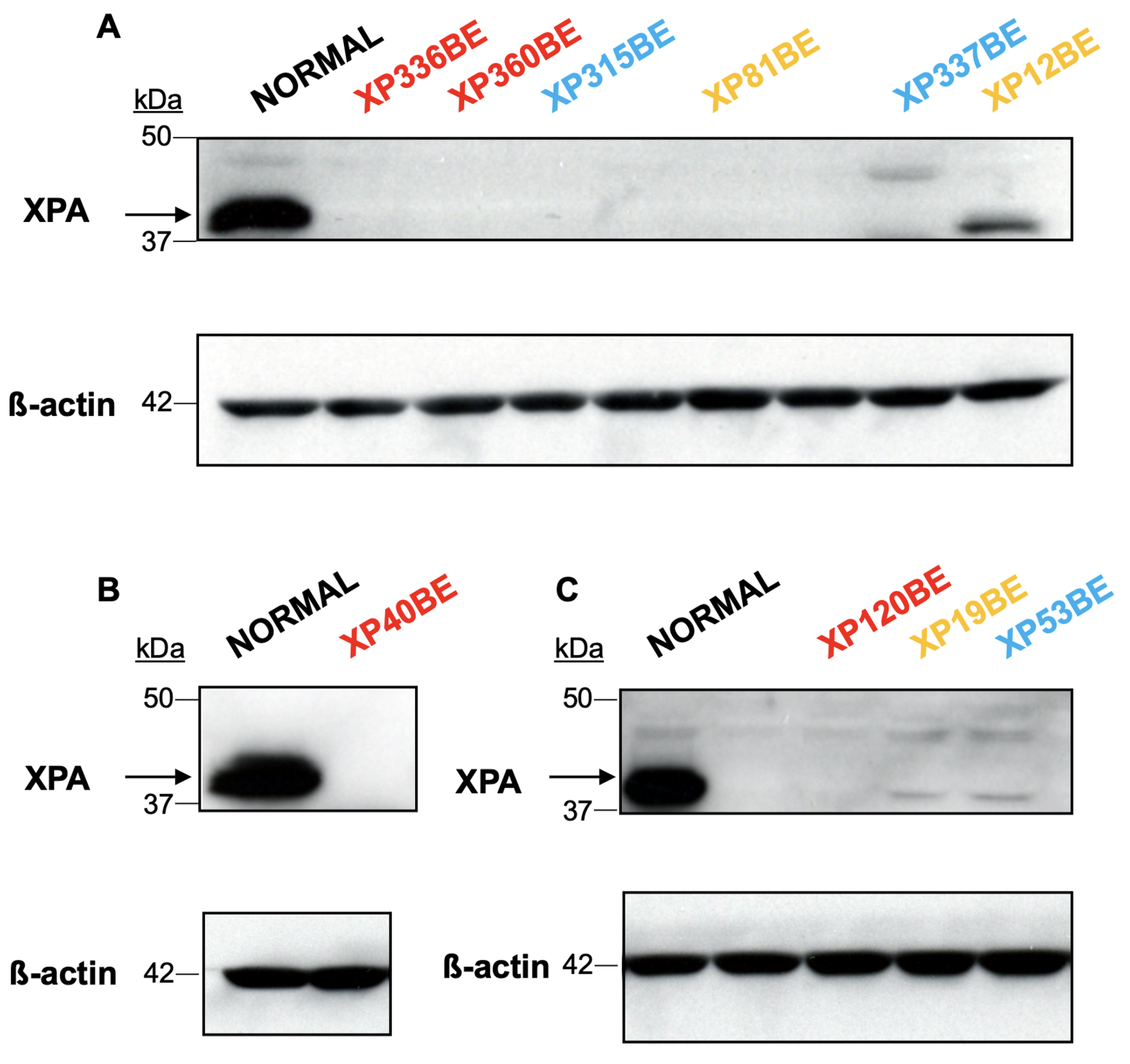

Supplement: S3 Fig — Western blotting was performed on extracts from fibroblasts and lymphoblasts probed with antibody for XPA (upper) and ß-actin (lower) in normal control, (A) XP336BE, XP360BE, XP315BE, XP81BE, XP337BE, XP12BE, (B) XP40BE, (C) XP120BE, XP19BE, and XP53BE. XPA protein indicated with arrows. XPA protein was reduced in XP12BE (31% of normal), XP19BE (4% of normal), and XP53BE (6% of normal). No detectable XPA protein in other XP-A patients (see methods section for details). (TIF) [file pgen.1011265.s003.tif]

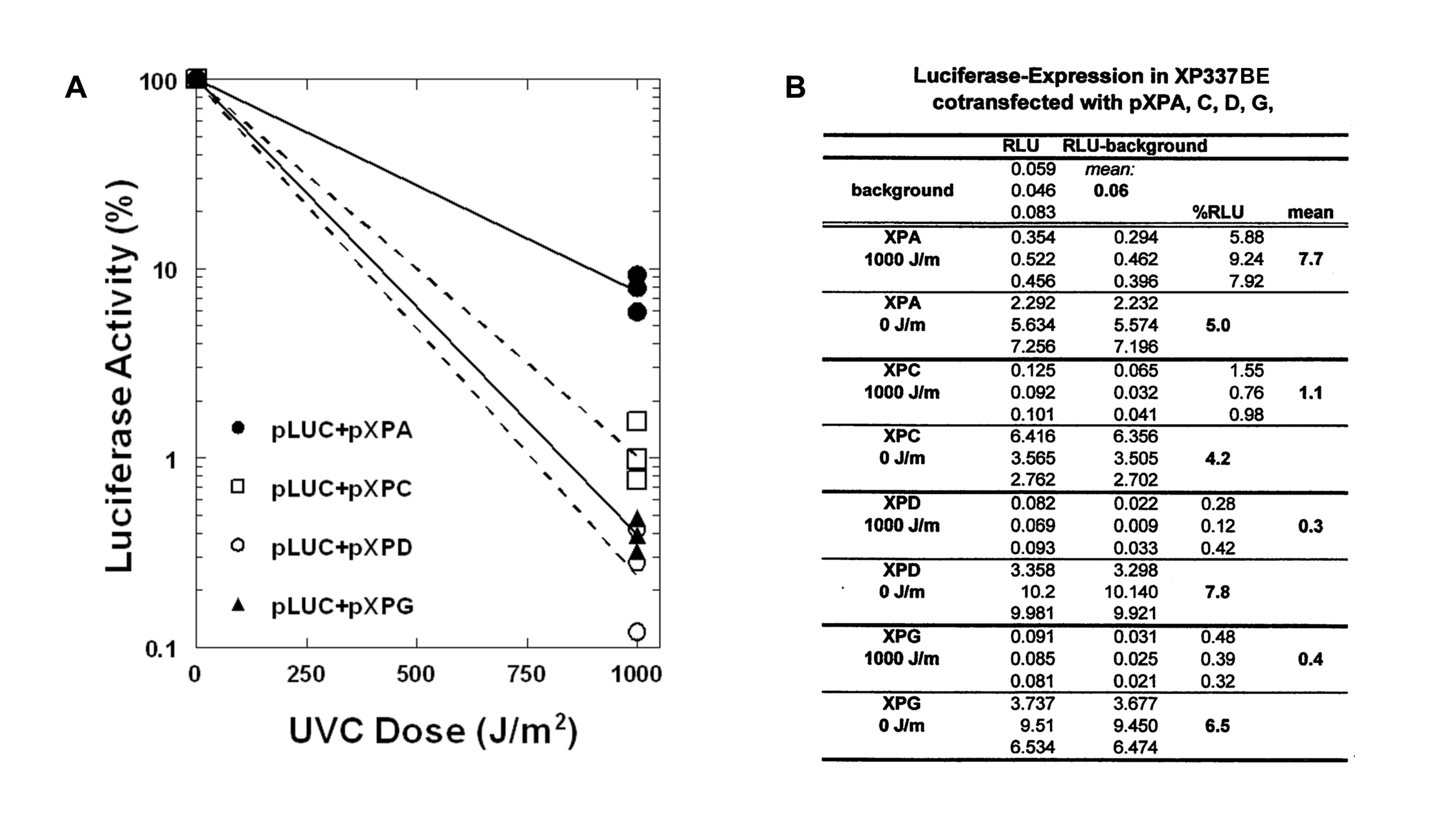

Supplement: S4 Fig — UV transfected reporter gene plasmid (pLUC) was co-transfected with XP complementary DNA containing plasmid [pXPA, pXPC, pXPD, and pXPG] into fibroblasts from XP337BE (see methods section for details). The correction was achieved only by co-transfection of pXPA indicating that these cells are in XP-A (see methods section for details). (A) Graph. (B) Data. (TIF) [file pgen.1011265.s004.tif]
