# Supplementary material for: Different germline variants in the XPA gene are associated with severe, intermediate, or mild neurodegeneration in xeroderma pigmentosum patients
Source: PLoS Genet. 2024 Dec 2;20(12):e1011265. doi: 10.1371/journal.pgen.1011265 (PMC11637439; doi:10.1371/journal.pgen.1011265)
Supplement: S1 Table — aNeurological severity was assessed using our neurological abnormality scoring scale in Table 1. “Unknown” indicates patients under age 10 years or with insufficient information to classify. bNumber of patients classified. cAge of patients classified. UDS: unscheduled DNA synthesis D37: dose that results in 37% cell survival after UVC irradiation (DOCX) [file pgen.1011265.s005.docx]

| **Author, Year [Reference]** | **Number of Patients** | **Age Range (Years)** | **Homozygous or Compound Heterozygous** | **Allele 1,**  **Protein Change** | **Allele 2,**  **Protein Change** | **Functional Tests** | **Neurological Severity^a^** |
| --- | --- | --- | --- | --- | --- | --- | --- |
| Fassihi et al., 2016 + Sethi et al., 2016 [26,31] | 12 | 7-80 | Homozygous | c.555+8A>G  (INT 4) | - | UDS: 3-20% of normal | Mild (10)^b^[19-80y]^c^  Unknown (2)[7-8y] |
| Whitworth et al., 2016 [49] | 1 | 65 | Homozygous | c.555+8A>G  (INT 4) | - | - | Mild (1) |
| Sidwell et al.,  2006 [50] | 1 | 61 | Homozygous | c.555+8A>G  (INT 4) | - | UDS: 2-5% of normal | Mild (1) |
| This report | 2 (XP53BE, XP618BE) | 16-39 | Homozygous | c.555+8A>G  (INT 4) | - | UDS: 8.2% of normal  D_37_: 1.05 Jm^-2^ | Mild (2) |

**S1 Table.** Summary of XPA c.555+8A>G splicing founder variant in 16 Pakistani and Indian XP-A patients.

^a^Neurological severity was assessed using our neurological abnormality scoring scale in Table 1. “Unknown” indicates patients under age 10 years or with insufficient information to classify.

^b^Number of patients classified.

^c^Age of patients classified.

**UDS**: unscheduled DNA synthesis **D_37_**: dose that results in 37% cell survival after UVC irradiation
