# Supplementary material for: Different germline variants in the XPA gene are associated with severe, intermediate, or mild neurodegeneration in xeroderma pigmentosum patients
Source: PLoS Genet. 2024 Dec 2;20(12):e1011265. doi: 10.1371/journal.pgen.1011265 (PMC11637439; doi:10.1371/journal.pgen.1011265)
Supplement: S2 Table — aNeurological severity was assessed using our neurological abnormality scoring scale in Table 1. “Unknown” indicates patients under age 10 years or with insufficient information to classify. bNumber of patients classified. cAge of patients classified. dOne intermediate patient died at age 35 years from neurological degeneration and multiple organ failure. eOne intermediate patient died at age 32 years from pneumonia fRef [27]. UDS: unscheduled DNA synthesis D37: dose that results in 37% cell survival after UVC irradiation LD50: median lethal dose RRS: recovery of RNA synthesis after DNA damage (DOCX) [file pgen.1011265.s006.docx]

| **Author, Year [Reference]**  **S2 Table.** Summary of XPA p.R228* nonsense founder variant in 49 North African XP-A patients. | **Number of Patients** | **Age Range (Years)** | **Homozygous or Compound Heterozygous** | **Allele 1,**  **Protein Change** | **Allele 2,**  **Protein Change** | **Functional Tests** | **Neurological Severity^a^** |
| --- | --- | --- | --- | --- | --- | --- | --- |
| Nishigori et al., 1993 [30] | 6 | 9-35 | Homozygous | c.682 C>T, p.R228* (EX 6) | - | UDS: 1-6% of normal  D_37_: 0.70-0.80 Jm^-2^ | Mild (1)^b^[35y]^c^, Intermediate (4)[15-34y], Unknown (1)[9y] |
| Kindil et al.,  2017 [64] | 7 | 7-33 | Homozygous | c.682 C>T, p.R228* (EX 6) | - | - | Intermediate (6)[12-33y], Unknown (1)[7y] |
| Sethi et al., 2016+Fassihi et al., 2016 [26,31] | 2 | 24-33 | Homozygous | c.682 C>T, p.R228* (EX 6) | - | UDS: 8% of normal | Intermediate (2) |
| Lehmann et al., 2015 [29] | 3 | 17-28 | Homozygous | c.747 C>T, p.R228* (EX 6) | - | LD_50_: 5 Jm^-2^ | Mild (3) |
| Messaoud et al., 2010 [62] | 10 | 0.13-26 | Homozygous | c.682 C>T, p.R228* (EX 6) | - | - | Intermediate (6)[12-26y], Unknown (4)[0.13-7y] |
| Maeda et al., 1995 [34] | 1 | 23 | Homozygous | c.682 C>T, p.R228* (EX 6) | - | D_37_: 0.55 Jm^-2^ | Intermediate (1) |
| Khalat et al., 2023 [63] | 2 | 18-21 | Homozygous | c.682 C>T, p.R228* (EX 6) | - | - | Mild (2) |
| Le May et al., 2018 [28] | 1 | 10 | Homozygous | c.682 C>T, p.R228* (EX 6) | - | UDS: 10% of normal  RRS: “failed” | Intermediate (1) |
| Bensenouci et al., 2016 [65] | 2 | 7-8 | Homozygous | c.682 C>T, p.R228* (EX 6) | - | - | Intermediate (2) |
| Soufir et al., 2010 [66] | 7 | Unknown | Homozygous | c.682 C>T, p.R228* (EX 6) | - | - | Mild (1), Intermediate (6) |
| Masaki et al., 2019 [95] | 2 | 35-48^d^ | Compound Heterozygous | c.682 C>T, p.R228* (EX 6) | IVS3-1G>C (INT 3) | - | Mild (1)[48y], Intermediate (1)[35y] |
| Maeda et al., 1995 [34] | 4 | 14-34^e^ | Compound Heterozygous | c.682 C>T, p.R228* (EX 6) | IVS3-1G>C (INT 3) | D_37_: 0.36-0.56 Jm^-2^ | Mild (1)[14y], Intermediate (3)[28-34y] |
| This report | 1 (XP591BE) | 16 | Compound Heterozygous | c.682 C>T, p.R228* (EX 6) | c.601_602delGA, p.E201Rfs*19 (EX 5) | - | Intermediate (1) |
| Garcia-Carmona et al., 2021 [27] | 1 | 14 | Compound Heterozygous | c.682 C>T, p.R228* (EX 6) | c.553C>T,  p.Q185* (EX 4) | UDS: 3.0% of normal | Intermediate (1) |

^a^Neurological severity was assessed using our neurological abnormality scoring scale in Table 1. “Unknown” indicates patients under age 10 years or with insufficient information to classify.

^b^Number of patients classified.

^c^Age of patients classified.

^d^One intermediate patient died at age 35 years from neurological degeneration and multiple organ failure.

^e^One intermediate patient died at age 32 years from pneumonia.

**UDS**: unscheduled DNA synthesis **D_37_**: dose that results in 37% cell survival after UVC irradiation **LD_50_**: median lethal dose **RRS**: recovery of RNA synthesis after DNA damage
